# Male Reproductive Phenotype Heterogeneity in Varicocele: A Hypothesis‐Generating Threshold‐Resilience Framework and Research Agenda

**DOI:** 10.1002/rmb2.70102

**Published:** 2026-10-15

**Authors:** Aris Kaltsas, Nikolaos Sofikitis, Michael Chrisofos

**Affiliations:** ^1^ Third Department of Urology, Attikon University Hospital, School of Medicine National and Kapodistrian University of Athens Athens Greece; ^2^ Department of Urology, Faculty of Medicine, School of Health Sciences University of Ioannina Ioannina Greece

**Keywords:** antioxidants, DNA fragmentation, male infertility, oxidative stress, Varicocele

## Abstract

**Background:**

Varicocele is common in male infertility, yet many affected men retain conventional reproductive measures, and the anatomical grade is an imperfect proxy for phenotype. Established pathways explain injury more clearly than between‐person heterogeneity.

**Methods:**

This structured, critical narrative review searched PubMed from 1 January 2015 to 26 August 2026, with targeted hand‐searching. There was no preregistered protocol, dual screening, or risk‐of‐bias assessment, and coverage is not exhaustive.

**Main Findings:**

The threshold‐resilience framework is a hypothesis‐generating model separating lesion‐related exposure, candidate host response measures, and couple‐level variables. Human varicocele‐specific evidence supports hemodynamic, thermal, redox, and sperm DNA damage associations. No prospective human varicocele study was identified in which an independently measured antioxidant, DNA processing, heat response, or endocrine measure preserved fertility at a given exposure. Female age and ovarian reserve are couple‐level variables, and all candidate host response measures remain research‐only.

**Conclusion:**

Intended for investigators in andrology and reproductive medicine, the framework is a research agenda rather than an explanatory mechanism or clinical algorithm. It maps evidence provenance, identifies measurement gaps, and specifies prospective tests of whether prespecified, outcome‐independent host response measures add prediction beyond anatomy, semen analysis, damage measures, and couple‐level factors.

## 1 Introduction

### 1.1 The Clinical Paradox: A Common Lesion, an Inconsistent Phenotype

Varicocele—the abnormal dilation of the pampiniform venous plexus—is among the most prevalent andrological findings in adult men, affecting approximately 15% of the general male population and occurring more often among men evaluated for fertility [1, 2, 3, 4]. It is a recognized, potentially correctable contributor to male infertility [5, 6], but its clinical expression is heterogeneous [7]. Clinical varicocele is also observed in male partners of ongoing unassisted pregnancies. In that selected cohort, a varicocele was associated with smaller left testicular volume, lower total and total motile sperm counts, and higher gonadotropins, whereas the unadjusted time to the study pregnancy was similar; ovarian‐reserve measures were not modeled [8]. An achieved couple pregnancy does not establish a preserved intrinsic male capacity independent of the female partner.

This discordance extends to treatment. Varicocele repair can improve semen parameters, serum testosterone, and pregnancy outcomes in appropriately selected couples, but semen gains do not reliably translate into spontaneous conception, and a meaningful subset of men derives little benefit [5, 7]. Anatomical presence is therefore an imperfect predictor of reproductive outcomes. This observation motivates the prospective study of phenotype heterogeneity but does not identify its cause.

In a tertiary‐clinic cross‐sectional survey restricted to men classified as having male‐factor infertility, a palpable varicocele was more prevalent among men with secondary than primary infertility (81% vs. 35%) [9]. Partners older than 40 years were excluded, but female age differed between groups, female infertility was only partly assessed, and no female‐variable adjustment was performed. An earlier cross‐sectional comparison also reported differences in testicular size among volunteers and subfertile clinic groups [10]. These findings are compatible with, but do not demonstrate, progressive male decline because neither study followed individual men longitudinally.

### 1.2 Limitations of a Deterministic “Varicocele → Bamage → Infertility” Model

The prevailing conceptual model treats varicocele as a linear cause: venous reflux produces a testicular insult, the insult produces damage, and the damage produces subfertility. Oxidative stress is now well established as the central convergent mechanism of that insult, arising from scrotal hyperthermia and testicular hypoxia, with the blood–testis barrier disruption acting downstream as an amplifying rather than an initiating step [7]. A strictly deterministic pathway, however, cannot accommodate the clinical reality. It does not explain why men with identical grades diverge so widely in phenotype, why subclinical lesions occasionally coincide with impairment, or why repair benefits some men and not others; no single mechanistic theory has proved adequate to account for this differential effect [5, 11].

Men with a similar anatomical grade may have different biological phenotypes, but current evidence does not establish that outcome‐independent host factors cause this discordance. The framework is used here only to organize candidate explanations for prospective testing.

### 1.3 Aim, Scope, and Rationale for an Integrative Framework

This narrative review proposes a hypothesis‐generating threshold‐resilience framework for varicocele‐associated male phenotypes. It distinguishes measured lesion‐related exposure from candidate host response variables provisionally grouped as susceptibility and putative male‐intrinsic reserve; neither construct is yet validated as an outcome‐independent determinant. Resilience denotes an observed longitudinal phenotype, not an inferred mechanism. The research hypothesis is that prespecified host response measures may be associated with male biological outcomes among men with comparable measured exposure; pregnancy and live birth must be analyzed separately at the couple level. Figure 1 depicts this proposed association.

**FIGURE 1:**
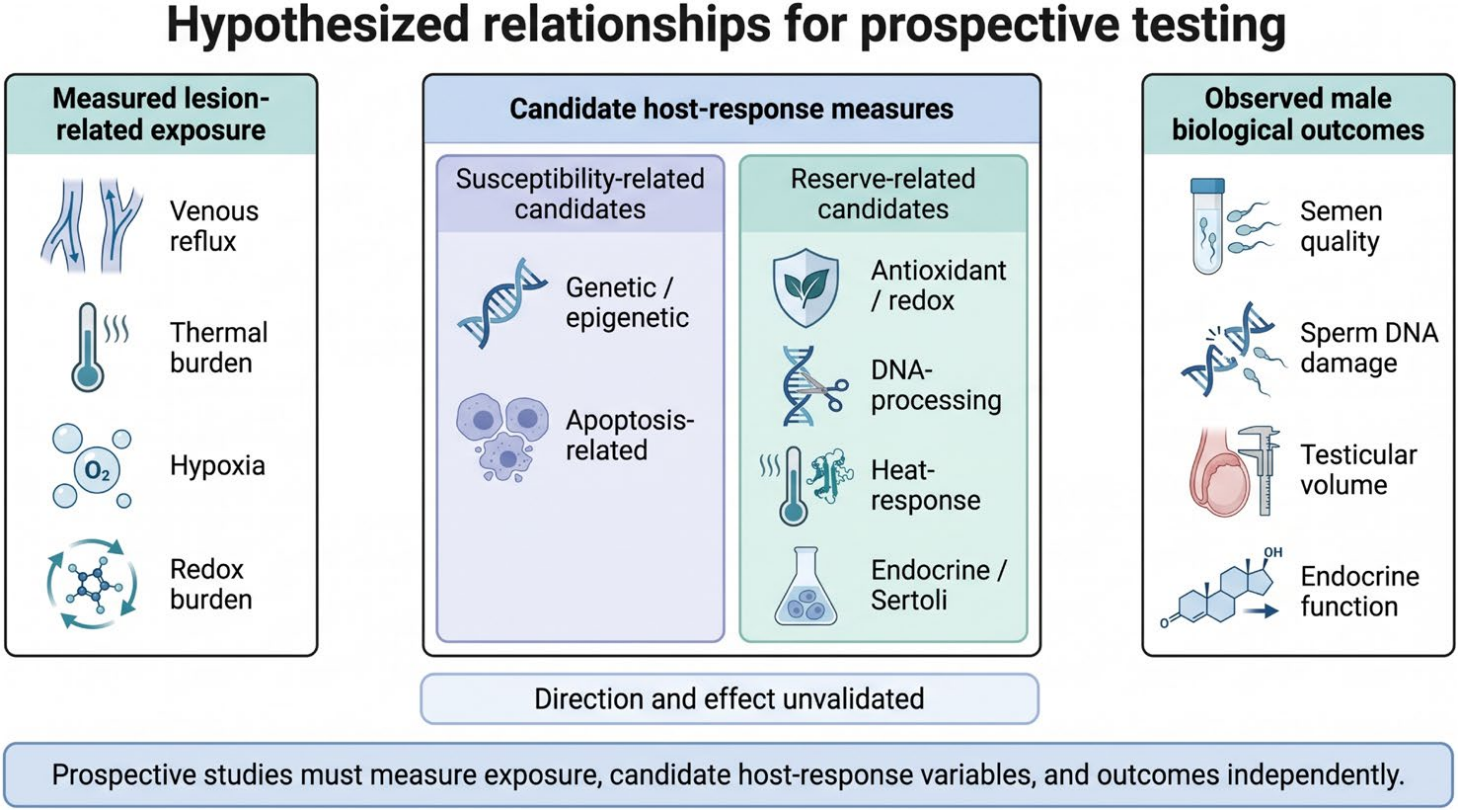
Schematic research hypothesis relating measured varicocele exposure, candidate host response measures, and male biological outcomes. The three groups are shown without directional links; the relationships between them are hypotheses for prospective study. The diagram does not establish that susceptibility amplifies injury, that reserve buffers it, that a critical threshold exists, or that treatment benefit is greatest near such a threshold. These propositions require prospective testing with outcome‐independent measurements. DNA, deoxyribonucleic acid. Created in BioRender. Kaltsas, A. (2026) https://BioRender.com/pf4fpyr.

This review is intended for andrologists, reproductive urologists, reproductive endocrinologists, embryologists, and investigators designing prognostic studies. Its scope is conceptual and research‐oriented: it organizes candidate exposure, host response, and couple‐level variables, identifies where current evidence is direct or indirect, and specifies falsifiable prospective tests. It does not provide a clinical decision algorithm or recommend the use of research‐only markers.

The framework proposes no new molecular pathway and does not replace the oxidative‐stress paradigm, which remains the principal convergent mechanism linking reflux‐related hyperthermia, hypoxia, and inflammatory or microenvironmental disturbances to testicular injury. Nor is inter‐individual variability a new observation: a recent mechanistic review notes wide variation in clinical presentation and reproductive outcomes [12]. The contribution proposed here is an organizational, hypothesis‐generating structure. It separates measured lesion‐related exposure, prespecified candidate host response measures, and couple‐level variables for analysis; the traditional susceptibility and reserve terms are retained only as provisional biological groupings and are not assumed to be distinct or measurable constructs. Candidate measures must be defined before the outcome, and their incremental prediction and exposure‐by‐prespecified‐measure interactions should be compared with conventional clinical and redox models. This review did not identify a prior synthesis combining these requirements with explicit refutation criteria in the same conceptual structure; this is a scoped claim about organization, not a claim of a new biological mechanism.

### 1.4 Evidence Acquisition, Selection, Appraisal, and Methodological Limitations

This structured critical narrative review was developed with reference to the SANRA quality criteria for narrative reviews [13]. PubMed was the sole bibliographic database searched. For the present revision, all queries were rerun on August 26, 2026 and covered records published from January 1, 2015 through that date, without a language filter. Table S1 reproduces the complete queries as entered, including the publication‐date limits, execution date, and update‐screening counts. Reference lists of retained reports and the websites or linked records of the AUA/ASRM, EAU, WHO, and Global Andrology Forum were hand‐searched, most recently on August 26, 2026. Backward citation searching was used to identify foundational reports published before 2015.

Primary human varicocele studies were eligible when they informed at least one stated evidence domain, including male reproductive phenotype, biomarkers, treatment response, pregnancy, or live birth. Evidence syntheses, relevant narrative reviews, and guideline documents were retained for context or clinical‐boundary statements. Human evidence from non‐varicocele populations was used only where direct varicocele evidence was unavailable and was labeled E3; animal or in vitro studies were used only to illuminate a specified mechanistic gap and were labeled E4; couple‐level studies were confined to the questions in Section 5. Records that did not inform a stated domain were not retained, and duplicate citations returned by multiple queries were considered once. Records were not intended to be selected according to whether their findings supported the framework; however, the absence of a prospective screening log for the initial search prevents independent reconstruction of all initial selection decisions. The August 26, 2026 update used a single‐reviewer record‐level screen with a PMID‐level ledger reported in Table S1. Screening was not duplicated independently and there was no adjudication process.

The initial evidence acquisition was iterative and narrative. De‐duplicated retrieval totals, full‐text exclusion logs, and reasons for exclusion were not retained prospectively and cannot be reconstructed reliably. No study‐level risk‐of‐bias instrument or certainty‐of‐evidence framework was applied. The E1–E5 categories indicate source and directness only; they do not grade methodological quality, risk of bias, or certainty. Reliance on one database, single‐reviewer selection, and the absence of prospective screening records and formal risk‐of‐bias assessments mean that selective citation, missed eligible evidence, and design‐specific bias cannot be excluded. The synthesis is therefore neither systematic nor claimed to be comprehensive or exhaustive and should be read as a critical conceptual appraisal.

For the claim‐level audit, one claim unit was defined as one tier‐homogeneous proposition cluster within a paragraph, figure legend, or table row; a block containing propositions supported by different tiers contributed one unit to each represented tier, whereas methods, administrative text, recommendation‐only text, and proposed study designs were excluded. The full census, which also covers the manuscript tables, comprised 211 claim units: E1 25 (11.8%), E2 73 (34.6%), E3 19 (9.0%), E4 19 (9.0%), and E5 75 (35.5%); E4 and E5 together accounted for 94 (44.5%). The narrative‐only census (Abstract through Conclusions, including legends but excluding table cells) comprised 139 units: E1 19 (13.7%), E2 40 (28.8%), E3 11 (7.9%), E4 9 (6.5%), and E5 60 (43.2%); E4 and E5 together accounted for 69 (49.6%). In the table‐inclusive census, the absolute E4 and E5 counts rose from 18 to 19 and from 71 to 75, while their combined share fell from 44.9% to 44.5%. The increase reflects propositions now labeled explicitly as animal, in vitro, or conceptual, not new speculative content. In the narrative‐only census, the E5 count fell from 64 to 60. The table of proposed studies was excluded because it reports research proposals rather than empirical claims. The coding rules, the excluded categories, and the distributions before and after this revision are reported in Table S3.

## 2 Varicocele as a Graded, Multifactorial Testicular Insult

Varicocele does not act through a single lesion‐specific mechanism but through a cluster of interacting derangements that share two features central to this review: they are convergent—funneling into a common oxidative endpoint—and they are dose‐graded rather than binary, scaling with reflux burden and exposure duration. The graded‐injury evidence defines the exposure side of the research model; whether prespecified host response measures account for additional outcome variation remains untested. Figure 2 separates established injury pathways from candidate measures proposed for prospective evaluation.

**FIGURE 2:**
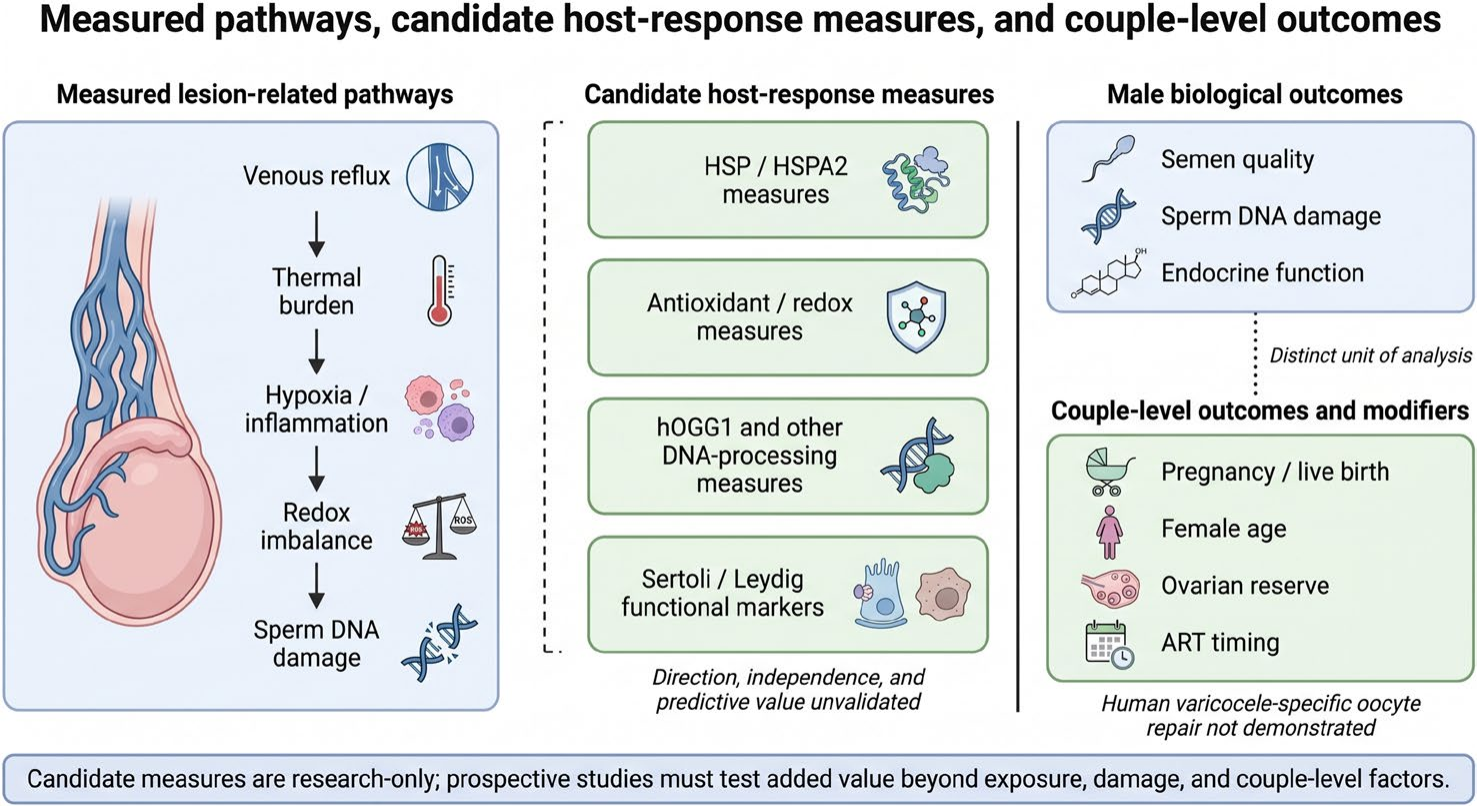
Measured lesion‐related pathways, candidate host response measures, and distinct male and couple‐level outcomes. Directional arrows are confined to the supported injury cascade; candidate measures are linked non‐directionally because their independence, direction, and predictive value remain unvalidated. Pregnancy and live birth are couple‐level endpoints and are separated from male semen, sperm DNA, and endocrine outcomes. Female age, ovarian reserve, and assisted reproduction timing are couple‐level variables; human varicocele‐specific oocyte‐mediated repair has not been demonstrated. All candidate measures remain research‐only. ART, assisted reproductive technology; DNA, deoxyribonucleic acid; hOGG1, human 8‐oxoguanine DNA glycosylase 1; HSP, heat‐shock protein; HSPA2, heat‐shock protein family A member 2; ROS, reactive oxygen species. Created in BioRender. Kaltsas, A. (2026) https://BioRender.com/pf4fpyr.

### 2.1 Venous Reflux, Stasis, and the Hemodynamic Basis

The initiating event is hemodynamic: incompetent or absent valves in the internal spermatic vein, together with impaired venous drainage, produce retrograde reflux and stasis within the pampiniform plexus [7, 14]. Three classic, non‐exclusive mechanisms link this venous derangement to testicular injury: elevated testicular temperature from the loss of counter‐current heat exchange, altered testicular blood flow with raised venous pressure, and the reflux of renal and adrenal metabolites down the incompetent vein [14, 15]. Each of these routes converges on the increase in parenchymal oxidative stress that underlies varicocele‐associated testicular dysfunction [7]. These observations support treating lesion‐related exposure as continuous rather than binary; they do not establish a host response term or a critical threshold.

### 2.2 Scrotal/Testicular Hyperthermia

Loss of efficient counter‐current cooling raises intrascrotal and intratesticular temperature, and spermatogenesis is acutely temperature‐sensitive; hyperthermia is thus a graded insult whose severity depends on the degree of thermal dysregulation [7, 16, 17]. In men with varicocele, continuous 24‐h monitoring confirms elevated scrotal temperature that correlates with impaired sperm parameters and higher follicle‐stimulating hormone (FSH), and the pattern is itself graded: normozoospermic men show a left‐sided temperature rise only, whereas oligozoospermic men show bilateral elevation [18]. In experimental systems, heat stress is accompanied by testicular injury and activation of heat‐response pathways [19]; these data do not show that individual thermotolerance modifies fertility in men with varicocele.

### 2.3 Hypoxia and HIF‐Axis Activation

Venous stasis impairs testicular perfusion and generates relative hypoxia, stabilizing hypoxia‐inducible factor 1‐alpha (HIF‐1α). In men with varicocele, HIF‐1α protein is increased in the internal spermatic vein [20], and HIF‐1α mRNA in seminal plasma is higher in infertile than in fertile affected men [21]. HIF‐1α acts as a hub, coupling hypoxia to germ‐cell apoptosis and sperm DNA fragmentation through downstream effectors including Bax and caspase‐3 [21]. In the cited animal model, autophagy and apoptosis indices increased, and Johnson scores decreased with longer experimental‐varicocele duration [22]. These animal associations do not establish a human apoptotic set‐point or show that apoptosis‐related measures determine individual susceptibility.

### 2.4 Oxidative Stress as the Convergent Final Common Pathway

Hyperthermia, hypoxia, and microenvironmental disruption converge on a single biochemical endpoint: a shift in the redox balance toward an excess of reactive oxygen species (ROS) relative to antioxidant capacity [23]. This convergence is what makes oxidative stress the mechanistic center of gravity of varicocele pathophysiology. A low, physiological concentration of ROS is required for normal sperm function—capacitation, hyperactivation, and the acrosome reaction—whereas supraphysiological ROS drives lipid peroxidation, sperm DNA damage, protein oxidation, enzyme inactivation, and apoptosis [23, 24]. In varicocele specifically, oxidative stress, frequently compounded by inflammation, degrades semen parameters, spermatogenesis, and testicular and epididymal function [25, 26, 27], and constitutes the principal upstream driver of the sperm DNA fragmentation cascade that links the lesion to impaired reproductive outcomes [28, 29].

Two properties of this pathway are pivotal for the framework. First, oxidative stress is graded: the degree of redox imbalance scales with the intensity of the upstream insults. Second, it is quantifiable—through seminal ROS assays, oxidation‐reduction potential (ORP) measured by the MiOXSYS system, oxidative DNA damage markers such as 8‐OHdG, and complementary assays of antioxidant capacity and sperm DNA fragmentation [24, 30, 31, 32, 33]. These continuous redox and damage measures are candidates for quantifying lesion‐related exposure and downstream injury in prospective tests; they do not operationalize reserve or establish a threshold.

### 2.5 Endocrine Perturbation (Paracrine and Systemic)

The insult is not confined to the germinal epithelium. Varicocele represents a pantesticular process that can impair Leydig cell steroidogenesis, lowering serum—and, by inference, intratesticular—testosterone, with partial reversibility of androgen production reported after varicocelectomy [34, 35]. The wider endocrine picture, however, is genuinely heterogeneous and must be presented with appropriate caution: across studies, findings range from reduced testosterone with a compensatory elevation of FSH and luteinizing hormone (LH) that normalizes after repair to reduced dihydrotestosterone, to lower circulating inhibin B and anti‐Müllerian hormone (AMH) interpreted as markers of diminished Sertoli cell function; results are inconsistent, and no uniform hormonal signature has been established [1, 36]. The same hormonal axes are candidate functional readouts for future studies; they are not validated measures of compensatory reserve.

These processes constitute a graded, multifactorial insult. Whether measured host response variables account for additional variation in clinical expression is an untested hypothesis.

## 3 Candidate Host Response Correlates Provisionally Grouped as Susceptibility

Within the proposed framework, “susceptibility” is a provisional label for prespecified, outcome‐independent host variables that may be associated with damage at a given measured exposure. A conversion ratio and an individual threshold have not been estimated in humans. The candidate variables below are grouped by biological rationale; their direction and incremental association with outcome require prospective testing.

### 3.1 Redox Heterogeneity and Candidate Baseline Measures

Excess seminal ROS has been reported in approximately 30%–80% of infertile men [24], demonstrating heterogeneity in redox measurements but not an individual buffering set‐point. The framework therefore treats the baseline redox balance as a candidate host response variable whose association with damage should be tested at a comparable measured varicocele exposure. No quantified individual redox set‐point has been measured in a varicocele. Evidence status: E5—conceptual inference.

### 3.2 Antioxidant‐Enzyme Genotypes as Candidate Correlates

Glutathione S‐transferase polymorphisms have been associated with semen and redox measures in men with idiopathic infertility [37]. These data are not varicocele‐specific. Varicocele‐specific GST findings are inconsistent [38, 39, 40]. GST measures are therefore candidate human correlates for prospective study; current evidence does not establish their direction or show that they explain unequal damage among men with comparable measured exposure. Evidence status: E3 for the indirect human association and E2 for the inconsistent varicocele‐specific associations; any susceptibility interpretation remains E5.

### 3.3 DNA‐Processing Genotypes and Sperm Epigenetic Correlates

In a single human varicocele cohort, the hOGG1 Ser326Cys Cys/Cys genotype was associated with higher sperm 8‐OHdG content, a lower mitochondrial DNA copy number, lower seminal TAC, and subfertile status [41]. Although hOGG1 participates in base‐excision repair, this observational association does not measure individual DNA‐repair capacity, establish temporality, or demonstrate effect modification among men with comparable measured exposure. hOGG1‐related measures are therefore candidate host response correlates for prospective study, not validated susceptibility or reserve markers.

Sperm epigenetic measures form a second candidate host response domain. Human spermatozoa from men with varicocele have shown global DNA hypomethylation relative to fertile controls, with differentially methylated regions mapping to gamete‐generation and meiotic pathways, and the magnitude of change reported to vary with varicocele grade [42, 43]. In an animal model, TET2 expression and 5‐hmC abundance changed alongside sperm quality measures [44]. These findings identify correlated molecular patterns; they do not show a pre‐existing or heritable susceptibility, establish a temporal direction, or demonstrate modification of human outcomes at a given exposure. Evidence status: E2 for human sperm‐methylation associations and E4 for the animal TET2 findings; any susceptibility interpretation remains E5.

### 3.4 Apoptosis‐Related Measures

Altered testicular expression of apoptosis‐regulating proteins (Bcl‐2, Bax, and caspases) has been described in men with varicocele [45, 46]. In an experimental animal model, apoptosis‐related indices changed with lesion duration while histological scores declined [22]. These observations motivate apoptosis‐related measures as candidate susceptibility variables, but they do not establish an individual apoptotic threshold or its direction of association with human outcomes. Evidence status: E2 for descriptive human expression findings and E4 for the animal‐model observations; any individual‐threshold interpretation is E5.

### 3.5 Age, Comorbidity, and Exposure Duration as Covariates

Finally, host vulnerability may change over time. Exposure duration belongs to the lesion‐related insult rather than to susceptibility or reserve; experimental evidence shows that hypoxic and apoptotic injury increases with lesion duration [22]. Advancing age and metabolic or inflammatory comorbidity should be treated as prespecified covariates. Dedicated varicocele‐specific human evidence has not established whether, or in what direction, they modify damage at a given exposure. It is also essential to distinguish high susceptibility from independent andrological disease. Coincident causes such as Y‐chromosome microdeletions, Klinefelter syndrome, a history of cryptorchidism, or gonadotoxin exposure are separate pathologies and must not be recorded as reserve failure [5]. Seminal oxidative‐stress readings should be interpreted only after leukocytospermia and genital‐tract infection have been excluded [23, 24, 28]. Attributing such cases to depleted reserve without first excluding these conditions would misclassify them within the framework. Evidence status: E4 for experimental duration findings; E5 for any age/comorbidity modifier interpretation in varicocele.

These candidate variables provide a conceptual grouping for prospective analysis. Current evidence does not show that they determine damage at a given exposure or that an opposing reserve term determines whether damage remains below a threshold.

## 4 Candidate Host Response Measures Provisionally Grouped as Putative Reserve

Compensatory reserve is used here as a proposed latent construct, not an observed cause of preserved fertility. Candidate host response measures are grouped by antioxidant, DNA processing, heat response, and endocrine domains. The testable hypothesis is that prespecified, outcome‐independent measures may be associated with maintained male function at comparable measured exposure; this has not been demonstrated. Susceptibility and reserve are provisional conceptual groupings that are not yet separately identifiable in existing data. Resilience denotes the observed longitudinal phenotype of maintained or recovered function, not an inferred mechanism. Female age, ovarian reserve, and assisted‐reproduction context are treated separately as couple‐level variables in Section 5.

### 4.1 Antioxidant and Redox Measures

Antioxidant capacity is an established component of redox biology and a candidate host response measure; available varicocele data do not validate it as a reserve construct. Seminal plasma is rich in enzymatic and non‐enzymatic antioxidants that protect spermatozoa from ROS, and physiological fertilization depends on maintaining ROS within a narrow window rather than eliminating it entirely [24, 47]. Oxidative stress becomes pathogenic when ROS generation outstrips antioxidant defense [23], and where that defense is exhausted, lipid peroxidation, DNA damage, and apoptosis ensue [23, 24]. Seminal total antioxidant capacity (TAC) is a measurable, state‐dependent antioxidant readout, but it should not be equated with reserve: greater antioxidant activity may reflect effective buffering, insufficient compensation, or a maladaptive reductive stress state, so its interpretation requires concurrent redox and functional measurements [31, 48, 49]. The postoperative antioxidant trials measured male semen or sperm DNA fragmentation endpoints only and did not assess pregnancy or live birth [50, 51]; they therefore support, at most, an association with a male laboratory response. A meta‐analysis found no clear improvement in pregnancy or semen outcomes in studies that had not screened for oxidative stress and called for trials restricted to men with documented oxidative stress [52]. Evidence status: E2 for human varicocele‐specific biomarker and intervention data; any interpretation of TAC as an outcome‐independent reserve measure is E5.

Human sperm‐proteomic evidence complicates the assumption that greater antioxidant activity necessarily represents greater reserve. In infertile men with unilateral varicocele, the upregulation of oxidant‐detoxification, glutathione, and NADH‐related pathways coexisted with impaired oxidative phosphorylation and altered heat stress responses. The investigators proposed that chronic reductive stress could itself contribute to sperm dysfunction [53]. Thus, the activation of antioxidant machinery may represent effective buffering, insufficient compensation, or a maladaptive reducing state; expression intensity alone cannot distinguish among these possibilities.

### 4.2 DNA Processing Measures

Oxidative sperm DNA lesions differ in type and biological consequence, and mature spermatozoa have limited capacity to complete repair: single‐strand breaks, typically oxidative and distributed across the genome, differ from double‐strand breaks linked to failed meiotic repair, so a fragmentation index depends on lesion type as well as quantity [54]. In one varicocele cohort, the hOGG1 genotype was associated with sperm 8‐OHdG, the mitochondrial DNA copy number, and seminal TAC [41]. This supports including DNA processing measures as candidate correlates, but no pre‐outcome capacity measure has predicted preserved fertility or treatment response; the proposed reserve interpretation remains unvalidated. The post‐fertilization contribution of the oocyte is a separate couple‐level research question (Section 5). Evidence status: E2 for a single human varicocele‐specific association study; E5 for a reserve or fertility‐prediction interpretation.

### 4.3 Heat Response Measures

Heat‐response biology is a candidate research domain. Experimental studies identify pathways involving eIF2α, IRE1α–XBP1, and endogenous antioxidant defenses [19], but they do not demonstrate a male reserve mechanism in human varicocele. Human varicocele‐specific evidence is limited but not absent. HSPA2 expression was reduced in adolescents with varicocele and oligozoospermia compared with both varicocele‐free controls and adolescents with varicocele and normal sperm concentration [55]. More recently, reduced HSPA2 abundance and delayed acrosomal localization were reported in samples from men with varicocele, with associations found for sperm motility and total motile sperm count [56]. These findings move HSPA2 beyond a purely preclinical candidate, but they remain cross‐sectional and do not demonstrate that independently measured thermotolerance capacity preserves fertility or predicts treatment response. Evidence status: E2 for human HSPA2 biomarker associations, E4 for experimental heat‐response mechanisms, and E5 for any independent reserve interpretation; prospective outcome validation is absent.

### 4.4 Leydig Cell Functional and Postoperative Response Measures

Many men with varicocele have serum testosterone within the reference range, and postoperative increases have been reported in a subset [34, 57, 58]. These observations do not identify a pre‐existing reserve because the putative measure is observed with or after the outcome. In one study, the postoperative change in 17‐hydroxyprogesterone was correlated with the change in total motile sperm count in hypogonadal men [59]. This is an outcome‐correlated response signal, not an independent reserve measure. Evidence status: E2 for the human hormone and postoperative‐response associations; E5 for any interpretation as pre‐outcome Leydig reserve.

### 4.5 Sertoli/BTB Research Domain

BTB disruption is described within the established varicocele injury literature [7]. BTB integrity and Sertoli‐related measures are candidate host response variables, but no dedicated human varicocele study has shown that either independently buffers injury or predicts preserved function. Endocrine markers provide indirect correlates of Sertoli function, not validated measures of reserve. Evidence status: E5 for any Sertoli/BTB reserve interpretation; no dedicated human varicocele reserve study was identified.

### 4.6 Endocrine Functional Correlates

Inhibin B, AMH, and FSH are accessible markers of Sertoli and endocrine function, with established physiological interrelationships [60]. In varicocele, reported AMH and inhibin B findings are inconsistent, and no uniform hormonal signature has been established [1, 61, 62]. These markers should be described as candidate functional correlates, not as a buffering system or readable index of reserve. The adolescent inhibin B and testicular‐volume findings are male developmental endpoints; adult fertility and couple outcomes were not measured [61]. Evidence status: E2 for the human biomarker findings and E5 for any outcome‐independent reserve interpretation.

Together, these measures form a candidate host response panel for study; Table 1 groups them provisionally by biological domain. Their aggregate value, between‐person distribution, association with outcome at matched exposure, and any relation to a threshold have not been established.

**TABLE 1:** Candidate host response measures are provisionally grouped by biological domain. The susceptibility‐related and reserve‐related columns are hypotheses for testing; they do not denote demonstrated directional effects or separately estimable constructs. Evidence types and limitations appear in the final column.

Biological domain	Candidate susceptibility‐related measures (direction unvalidated)	Candidate reserve‐related measures (direction unvalidated)	Evidence type caveat
Baseline redox measures	GST genotypes were associated with TAC, 8‐OHdG, MDA, and semen differences in indirect human cohorts [37]; direction and varicocele specificity remain uncertain [38, 39, 40]	Antioxidant measures evaluated relative to oxidative load; the direction of TAC association should not be assumed monotonic [23, 24, 31]	E3 for indirect human associations [37]; E2 for inconsistent varicocele‐specific associations [38, 39, 40]
DNA‐processing measures	hOGG1 Ser326Cys Cys/Cys was associated with higher sperm 8‐OHdG, lower mitochondrial DNA copy number, and lower TAC in one cohort [41]	hOGG1 and other DNA‐processing measures proposed for association testing; no validated outcome‐independent capacity [41, 54]	E2—hOGG1 human (varicocele), single cohort; post‐fertilization oocyte‐related effects remain a separate couple‐level hypothesis
Sperm epigenetic measures	Human sperm‐methylation differences and grade association [42]; animal TET2/5‐hmC changes observed alongside sperm‐quality measures [44]	Preserved methylation integrity—proposed correlate; association with outcome untested (E5)	E2 human [42]; E4 TET2 translational [44]; any pre‐existing host response interpretation is E5
Heat‐response measures	Heat sensitivity of spermatogenesis; graded scrotal‐temperature elevation [16, 18]	Experimental eIF2α/stress‐granule, IRE1α–XBP1, and HSP pathway measures; human HSPA2 abundance as a cross‐sectional correlate [19, 55, 56]	E2 for human temperature/HSPA2 findings [55, 56]; E4 for experimental pathways [19]; no prospective fertility validation
Apoptosis‐related measures	Apoptosis‐related measures observed in an animal model; human individual set‐point untested [22]	Apoptosis‐related measures—proposed host response correlates; human outcome association untested (E5)	E4—translational (rat) [22]; any human individual‐effect interpretation is E5
Steroidogenic (Leydig)	Lower testosterone reported in some human cohorts; endocrine findings are heterogeneous [34]	Observed maintenance or postoperative recovery of testosterone; postoperative 17‐OHP change is an outcome‐correlated response marker, not independent reserve [34, 59]	E2—human; the reserve reading is inferred from postoperative recovery and is therefore E5 (Section 6.5)
Sertoli/blood–testis barrier	BTB disruption as a route of oxidative injury to germinal epithelium [7]	BTB/Sertoli measures proposed for evaluation; no validated outcome‐independent reserve measure (E5)	E5—conceptual; embedded in mechanistic reviews, no dedicated varicocele data
Endocrine feedback measures	Inhibin B, AMH, and FSH differences reported; findings are inconsistent across studies [1]	Candidate Sertoli and endocrine measures for prospective association testing; no validated independent host response construct [1, 60]	E2—human; inconsistent across studies (no uniform signature)
Temporal & host modifiers	Exposure duration in an animal model [22]; age and metabolic/inflammatory comorbidity as prespecified human covariates with unestablished direction	Exposure duration and host covariates for prospective adjustment; direction and magnitude in humans not established	E4 for animal‐model duration findings; E5 for any human age/comorbidity modifier interpretation

Abbreviations: 5‐hmC, 5‐hydroxymethylcytosine; 8‐OHdG, 8‐hydroxy‐2′‐deoxyguanosine; AMH, anti‐Müllerian hormone; BTB, blood–testis barrier; eIF2α, eukaryotic initiation factor 2 alpha; FSH, follicle‐stimulating hormone; GST, glutathione S‐transferase; HIF‐1α, hypoxia‐inducible factor 1‐alpha; hOGG1, human 8‐oxoguanine DNA glycosylase 1; HSP/HSPA2, heat‐shock protein/heat‐shock protein family A member 2; IRE1α–XBP1, inositol‐requiring enzyme 1 alpha–X‐box binding protein 1; MDA, malondialdehyde; TAC, total antioxidant capacity; TET2, ten‐eleven translocation methylcytosine dioxygenase 2; 17‐OHP, 17‐hydroxyprogesterone.

## 5 Couple‐Level Modifiers of the Translation From Male Phenotype to Reproductive Outcome

Experimental work in a non‐mammalian model motivates the hypothesis that oocyte‐related factors may modify developmental outcomes after sperm DNA damage [63]. It does not demonstrate repair‐mediated rescue in human varicocele. Female age, ovarian‐reserve measures, infertility duration, and the assisted‐reproduction context should therefore be handled as measured couple‐level variables rather than as evidence of a compensatory mechanism or a component of male reserve.

In the cited experimental model, embryo‐development outcomes differed by sperm DNA damage burden and oocyte‐related conditions [64]. These results should not be generalized to a human or varicocele‐specific repair threshold. Indirect human ART studies associate sperm DNA damage with implantation, pregnancy, or live birth, but those are couple‐level outcomes, and the magnitude of association varies by treatment context [65, 66, 67].

Among non‐varicocele ART cohorts, female age and ovarian reserve altered observed associations between sperm DNA damage and couple outcomes. Simon et al. [65] reported lower implantation with a female age above 35 years but did not measure oocyte repair or isolate it from other female and embryology factors. Khalafalla et al. [68] reported higher clinical pregnancy and live‐birth rates in women younger than 35 years with an AMH of at least 7.1 pmol/L within a high‐SDF subgroup; this was a retrospective stratified analysis, not evidence of oocyte‐mediated repair. A varicocelectomy‐before‐ART meta‐analysis suggested greater benefit in studies with a lower mean female age, an ecological study‐level subgroup rather than a patient‐level adjustment [69]. These data support female age and ovarian reserve as couple‐level prognostic factors; they do not demonstrate that oocytes rescue varicocele‐associated sperm damage.

Observed pregnancy cannot be attributed to male reserve unless female‐partner factors and reproductive exposure are measured and modeled. Evidence status: E3 for indirect human ART associations and E4 for the oocyte‐repair mechanism; no direct human study identified in this review demonstrated oocyte‐mediated rescue of varicocele‐associated sperm damage. Table S2 reports the unit of analysis and female‐factor handling for each cited primary outcome study.

## 6 The Threshold‐Resilience Framework as a Prospective Research Structure

An integrative research framework can distinguish measured lesion‐related exposure, candidate host response measures, and couple‐level factors. It does not assert that host factors determine damage, that reserves offset it, or that a threshold exists. Resilience denotes an observed longitudinal phenotype rather than an inferred mechanism. The framework is offered to organize evidence and prespecify tests, not as an empirically fitted equation or explanatory balance.

### 6.1 Testable Association Statement

The framework poses the testable hypothesis that, among men with comparable measured lesion‐related exposure, prespecified outcome‐independent host response measures are associated with male biological outcomes.

A future model may test whether associations among measured exposure, candidate host response variables, and male biological outcomes are nonlinear and whether a reproducible change point exists; preservation below a critical threshold is not assumed.

Insult, susceptibility, reserve, and threshold are heuristic labels. Existing evidence supports the measurement of several lesion‐related exposure and damage variables, but current datasets do not identify a host multiplier, an aggregate buffering capacity, or a critical threshold. No causal functional form or quantitative specification is claimed. Because reserve is not independently measured in existing datasets, it must not be inferred post hoc from discordant outcomes. Prospective prespecification of candidate host response measures and explicit falsification tests are required before the framework can be evaluated as more than a descriptive research hypothesis.

The model should be considered at two linked but distinct levels. The male biological endpoint is a sustained change in semen quality, sperm DNA damage, testicular volume, or endocrine function. Pregnancy and live birth are downstream couple‐level endpoints and must be modeled separately, with female age, ovarian reserve, infertility duration, intercourse or assisted‐reproduction exposure, and the other couple‐level modifiers set out in Section 5. To avoid circularity, future studies must prespecify exposure, damage, host response measures, and outcomes; any candidate change point must be estimated and externally validated, not defined from the outcome it is claimed to explain.

### 6.2 Provisional Host Response Groupings and Empirical Non‐Identifiability

Susceptibility and reserve are proposed conceptual domains, but current data do not identify them as separate axes. No current evidence justifies assigning an individual to a two‐dimensional space or locating a critical threshold. ROS and ORP provide redox measures, while 8‐OHdG and sperm DNA fragmentation are downstream damage measures; TAC and endocrine markers are candidate host response correlates, not validated reserve measures. Table 2 summarizes the evidence provenance and explicitly lists the untested central E5 hypothesis.

**TABLE 2:** Evidence provenance across the threshold–resilience research framework. Each element is mapped to the applicable evidence tier or tiers using the E1–E5 scheme defined in Section 1.4. Biomarker associations are separated from any reserve interpretation. The central hypothesis—that independently measured host response measures are associated with preserved male function at a given exposure—is listed explicitly as E5 because it has not been tested.

Model element	Role	Evidence tier (E1–E5, Section 1.4)	Support
Outcome‐independent host response association with preserved male function	Central research hypothesis	E5—untested conceptual inference	No direct supporting primary study identified
Scrotal/testicular hyperthermia	Insult	E2—human, direct temperature measurement	[16, 17, 18]
Hypoxia/HIF‐1α activation	Insult	E2—human tissue and seminal plasma	[20, 21]
Oxidative stress (ROS, 8‐OHdG)	Insult (convergent)	E2—human, varicocele‐specific	[27, 32, 33, 47]
Antioxidant‐enzyme genotype (GST)	Candidate susceptibility correlate	E2 for inconsistent varicocele‐specific associations; E3 for non‐varicocele human associations; E5 for any directional susceptibility interpretation	[37, 38, 39, 40]
DNA‐repair genotype (hOGG1)	Candidate hOGG1‐associated correlate	E2—human, varicocele‐specific (single cohort)	[41]
Sperm DNA methylation/epigenetic state	Candidate sperm‐epigenetic correlate	E2 for human methylation associations; E4 for animal TET2 findings; E5 for any pre‐existing susceptibility interpretation	[42, 43, 44]
Apoptosis‐related measures	Candidate apoptosis‐related correlate	E4 for animal‐model apoptosis findings; human expression data are descriptive; E5 for any individual set‐point or susceptibility interpretation	[22, 45, 46]
Seminal antioxidant capacity (TAC)	Candidate redox/host response correlate; prospective outcome validation absent	E2 for TAC biomarker measurement; E5 for any reserve interpretation; prospective outcome validation absent	[48, 49]
hOGG1 and other DNA‐processing measures	Candidate DNA‐processing correlate	E2 for the single‐cohort genotype–biomarker association; E5 for any outcome‐independent reserve interpretation	[41, 54]
Oocyte‐related variables	Couple‐level variables and research question—not a male reserve mechanism	E4 experimental repair findings; E3 indirect human ART associations; no human varicocele‐specific repair‐mediated rescue demonstrated	[63, 68]
HSPA2 and other heat‐response measures	Candidate host response correlate	E2 for cross‐sectional human HSPA2 associations; E4 for experimental mechanisms; E5 for any reserve interpretation	[19, 55, 56]
Testosterone and postoperative steroidogenic response	Functional and outcome‐correlated response measures	E2 for postoperative hormone‐response associations; E5 for any pre‐outcome reserve interpretation	[34, 35, 59]
Sertoli/BTB measures	Proposed research domain	E5—no dedicated human varicocele study or validated outcome‐independent reserve measure	[7]
Endocrine feedback (inhibin B, AMH)	Candidate readout of functional capacity	E2—human, inconsistent; readout rather than demonstrated buffer	[1, 60, 61, 62, 70]
Documented couple reproductive history	Partner pregnancy or prior paternity; couple‐history endpoint	E1 for observed history; female factors incompletely modeled; no independent male‐capacity inference	[8, 71]
Author‐defined fertile/normozoospermic male comparator	Male semen or biomarker endpoint	E2; fertility definition or female‐factor handling unreported or unclear	[72, 73]
Incidental high‐grade varicocele with preserved conventional semen	Male endpoint only; no reproductive outcome	E2; no inference about pregnancy, live birth, or intrinsic male fertility	[74]

Abbreviations: 8‐OHdG, 8‐hydroxy‐2′‐deoxyguanosine; AMH, anti‐Müllerian hormone; ART, assisted reproductive technology; E1–E5, evidence‐source/directness tiers defined in Section 1.4; GST, glutathione S‐transferase; HIF‐1α, hypoxia‐inducible factor 1‐alpha; hOGG1, human 8‐oxoguanine DNA glycosylase 1; HSPA2, heat‐shock protein family A member 2; ORP, oxidation–reduction potential; ROS, reactive oxygen species; TAC, total antioxidant capacity; TET2, ten‐eleven translocation methylcytosine dioxygenase 2.

Current methods quantify the redox state and downstream damage more readily than they quantify proposed host response constructs. Oxidation‐reduction potential and reactive oxygen species reflect the redox state, whereas 8‐OHdG and sperm DNA fragmentation are downstream damage readouts. Total antioxidant capacity, testicular volume, FSH, and inhibin B are measurable correlates, but each is state‐dependent, and no validated instrument measures a distinct reserve construct. The framework is therefore not operationalized.

No validated instrument separates susceptibility from reserve, and existing data do not identify them as distinct axes. When only lesion‐related exposure and downstream outcome are measured, any unmeasured host contribution is non‐identifiable. Near‐term tests should ask whether prespecified host response measures add reproducible predictive information beyond conventional, redox, damage, and couple‐level variables. Where Tables 1, 2 use susceptibility‐ or reserve‐related groupings, they are provisional biological categories rather than empirically separable constructs.

### 6.3 Discordant Phenotypes: What the Framework Does and Does Not Explain

Phenotypic discordance is compatible with the framework but is not specific evidence for it. A man with high‐grade or bilateral disease and preserved semen may have favorable host biology, shorter cumulative exposure, measurement variability, or unmeasured couple‐level advantages; conversely, impaired semen in subclinical or low‐grade disease may reflect susceptibility, coincident male pathology, or the misclassification of lesion severity. The framework organizes these possibilities and generates testable hypotheses, but reserve must not be inferred from the observed outcome: demonstrating a reserve effect requires prospective, independent measurement before fertility or treatment response is known [5].

### 6.4 Treatment‐Response Hypotheses and Observed Latency

The framework generates, but current evidence does not test, a hypothesis that prespecified baseline host response measures may be associated with the response to varicocelectomy at comparable exposure and damage. Meta‐analyses report a reduction in DNA fragmentation after repair [75, 76, 77], and pooled temporal data describe an average change through approximately six months [78, 79]. These observations do not identify reserve, a critical threshold, preferential benefit near a threshold, or a reserve‐dependent treatment window; the observed plateau may reflect multiple alternative processes.

Persistence or recurrence is associated with continuing lesion‐related exposure and may limit the response [80]. It does not establish movement relative to a threshold or identify reserve. Patient selection should remain guideline based while candidate host response measures are evaluated prospectively.

### 6.5 Prospective Operationalization and Falsification

Prospective validation should prespecify lesion‐related exposure, downstream damage, candidate host response measures, and outcomes before follow‐up. A conventional baseline model should include age, clinical grade, laterality, semen parameters, infertility duration, and relevant couple‐level factors. A redox measure—seminal ORP or reactive oxygen species—should also be included because a model that outperformed only a clinical baseline would not distinguish the framework from the established oxidative‐stress paradigm. Candidate non‐redox measures may include testicular volume, FSH, and inhibin B, with HSPA2, hOGG1 status, and multi‐omic markers exploratory. Total antioxidant capacity should be evaluated separately because it is redox‐proximal and may be non‐monotonic. Components, transformations, and the analysis plan should be specified before follow‐up; model weights and any candidate change point may be estimated in a derivation cohort but must be locked and tested in external validation.

The primary test is whether prespecified, outcome‐independent host response measures add reproducible discrimination and calibration beyond conventional, redox, damage, and couple‐level variables. Secondary tests may examine exposure‐by‐measure interactions and nonlinear change points without assuming that either exists. Pregnancy and live birth require separate couple‐level models. The framework would be weakened or refuted by no incremental predictive value, no reproducible interaction or change point, or equivalent performance from exposure and conventional semen variables alone. A candidate measure must not be defined from, measured after, or fitted to the outcome it is claimed to explain.

Total antioxidant capacity should be modeled as an exploratory predictor with a prespecified flexible functional form. Competing monotonic and nonlinear models should be compared; available evidence does not support assigning a positive direction or interpreting TAC as reserve [53].

### 6.6 Alternative Explanations and Where the Framework Could Be Wrong

The framework should be tested against simpler rivals. In a large, unselected general‐population cohort, varicocele grade was associated with semen quality and reproductive hormone levels in a dose‐dependent manner [36], supporting a graded‐injury alternative. Differences between this pattern and infertility‐clinic series may partly reflect clinical ascertainment rather than host biology. The framework should be retained only if prespecified host response measures add reproducible information beyond the simpler model. Measurement noise in semen analysis, unmeasured female‐partner factors, and independent andrological disease must not be relabeled post hoc as an adverse host response. If adequately powered studies show no incremental predictive value after exposure, damage, conventional semen, and couple‐level variables are modeled, the central hypothesis would fail. None of these possibilities is currently excluded.

## 7 Evidence From Men With Preserved Semen or Documented Couple Reproductive History

Existing reports document distinct phenotypes that should not be conflated: prior paternity [71], inclusion as the male partner of an ongoing unassisted pregnancy [8], author‐defined fertile or normozoospermic comparator status [72, 73], and incidental high‐grade varicocele with normal conventional semen [74]. Only the first two contain a couple reproductive history, and neither establishes male fertility independent of female‐partner factors.

### 7.1 Prior Paternity, Partner Pregnancy, and Normozoospermic Comparator Cohorts

Study [71] shows that varicocele can coexist with prior paternity, but historical paternity does not measure current male capacity or future couple fecundity. Study [72] used an author‐defined fertile normozoospermic comparator without reporting operational fertility criteria or female‐partner factors and therefore supports a preserved semen/biomarker phenotype only. Study [73] reported no semen or ROS difference between author‐defined fertile men with and without clinical varicocele; no prospective reproductive outcome was measured, and female‐partner handling could not be verified. Study [74] reported generally normal sperm concentration and motility among men with incidental grade 2/3 lesions referred for military‐service grading; no fertility or pregnancy outcome was measured. The coexistence of varicocele with prior paternity, current partner pregnancy, or normozoospermia is documented, but these categories are not interchangeable evidence of intrinsic male fertility.

Among male partners of ongoing unassisted pregnancies, clinical varicocele was associated with lower male sperm output and smaller left testicular volume, while the observed unadjusted time to the study pregnancy was similar [8]. Pregnant partners of men with varicocele were slightly older, and ovarian‐reserve measures were not modeled. The study documents achieved couple fecundity alongside male laboratory differences; it does not establish a sub‐threshold state, biological containment, or a mechanism preserving intrinsic male fertility.

### 7.2 Incidental and Subclinical Varicocele

Most varicoceles are asymptomatic [7], but the absence of symptoms does not establish preserved fertility. Subclinical and low‐grade disease illustrate the incomplete relation between anatomy and function: in a cohort spanning subclinical through clinical grades, testicular volume and semen analysis parameters did not differ cleanly across grade categories, and management was linked more closely to fertility history, testicular atrophy, and abnormal semen than to anatomical grade alone [81]. Subclinical disease can still be associated with a measurable change in some men [82, 83, 84]. These findings show that anatomical severity is an imperfect proxy for the functional phenotype; they do not identify the source of the discordance.

### 7.3 Inter‐Individual Variability and Discordance Between Grade and Function

Clinical and ultrasonographic grades correlated reasonably well with each other, yet neither tracked reliably with semen quality or testicular volume [74, 81]. Because the grade is an imprecise surrogate for cumulative exposure and the semen analysis is intrinsically variable, discordance may arise from exposure misclassification, biological variability, coincident andrological disease, or couple‐level factors. The empirical question is whether prespecified host response measures add reproducible predictive information after these alternatives are addressed.

### 7.4 What Preserved‐Semen and Reproductive‐History Cohorts Do and Do Not Show

The coexistence of a varicocele with prior paternity, ongoing partner pregnancy, or preserved conventional semen is documented [8, 71, 72, 73, 74]. It does not show that these men are protected by a superior, independently measured reserve. Among the studies identified in this review, no longitudinal dyadic cohort measured candidate male host response markers before conception while prospectively modeling female age, ovarian reserve, infertility duration, and intercourse or assisted‐reproduction exposure. Cross‐sectional proteomic, miRNA, and metabolomic differences across semen‐defined varicocele phenotypes identify candidate correlates [85, 86, 87], but they cannot determine whether they precede the phenotype, represent an adaptive response, or reflect disease severity and duration.

## 8 Candidate Phenotyping Measures and Research Readiness

Current tools measure several lesion‐related exposure and damage correlates, whereas candidate host response measures remain poorly validated. This asymmetry motivates prospective research but has not been shown to explain the difficulty in patient selection. The following section distinguishes established clinical measures from candidate host response measures without treating them as validated model axes.

### 8.1 Grading (Dubin–Amelar), Subclinical Disease, and Functional Correlation

Clinical grading by the Dubin–Amelar system and its ultrasonographic counterparts (e.g., Sarteschi) is the standard first‐line phenotyping of the lesion, and clinical and color‐Doppler grades correlate reasonably well with one another [81]. As an index of insult load, however, the grade performs poorly, and the practical recommendation has been to let the fertility history, testicular atrophy, and abnormal semen—not anatomical grade alone—drive decisions [81]. Within the research framework, the grade is treated as an anatomical descriptor with limited standalone prognostic weight; it does not locate a patient on a threshold or establish any other model term.

### 8.2 Laterality and Bilaterality

Laterality is most coherently understood as a modifier of insult load rather than as an independent, deterministic driver of outcome, and two lines of evidence support this reading. First, the thermal consequence of a unilateral lesion is not reliably confined to the affected side: in men with a varicocele, the distribution of scrotal‐temperature elevation tracks semen quality, with normozoospermic men showing a left‐sided rise only and oligozoospermic men showing bilateral elevation [16, 18]. That comparison is cross‐sectional and cannot establish that contralateral heating precedes the semen abnormality; read conservatively, it shows that unilateral disease need not be a unilateral exposure, which is one reason side alone predicts outcome poorly. Second, where bilateral disease is present, more complete insult reduction yields better results: in a systematic review and meta‐analysis, bilateral varicocelectomy was superior to unilateral repair in men with bilateral (including left‐clinical/right‐subclinical) varicocele, with higher spontaneous pregnancy rates and greater gains in sperm parameters [88], a finding corroborated by randomized data [89] and consistent with bilateral repair featuring among the predictors of semen improvement in a systematic appraisal [90].

These observations support using laterality as an exposure‐related descriptor. They do not show that preserved function in bilateral disease is caused by a high reserve or that any man lies below a threshold. Clinical decisions about the extent of repair should remain tied to the applicable evidence and guidelines.

### 8.3 Semen, Sperm DNA Fragmentation, and Oxidative‐Stress Readouts

The insult–damage axis is where measurement is strongest. Three tiers of readout are available. Conventional semen analysis captures the downstream functional consequence but is an insensitive and variable proxy for the underlying molecular insult. Sperm DNA fragmentation (SDF), commonly quantified as a DNA fragmentation index (DFI), sits closer to the mechanism: it reflects the oxidative DNA‐damage endpoint of the insult cascade, meta‐analytic data confirm that the DFI decreases significantly after varicocelectomy, and it therefore functions both as a stratifier of current damage and as a monitor of response [28, 75, 91]. Oxidative‐stress readouts sit closest of all to the primary mechanism: seminal ROS and oxidation‐reduction potential measured by the MiOXSYS system provide direct, continuous indices of redox imbalance and thus most directly operationalize the model's insult axis [28, 30].

A static ORP threshold of approximately 1.36 mV/10^6^ sperm/mL was proposed in an early MiOXSYS study, with a sensitivity of 69.6% and a specificity of 83.1% [92]. It should not be interpreted as a universal reference or treatment threshold. A large multicenter evaluation applying a near‐identical cutoff of 1.34 mV/10^6^ sperm/mL reported markedly different operating characteristics—98.1% sensitivity but only 40.6% specificity [93]—whereas a later cohort derived a substantially lower cutoff of 0.79, with 57.7% sensitivity and 73.1% specificity, and questioned normalization to sperm concentration because concentration forms the denominator of the static ORP (sORP) index [94]. Similarly, DFI thresholds are assay‐specific: the sperm chromatin structure assay, TUNEL, the sperm chromatin dispersion test, and the Comet assay measure different lesion classes, do not yield interchangeable values, and are all affected by preanalytical conditions and methodology [28, 31], a limitation the guideline literature echoes in treating sperm DNA fragmentation as an emerging rather than an established biomarker [95]. ORP and SDF should therefore be interpreted as adjunctive, platform‐dependent measurements rather than standalone indications for varicocele repair [30].

### 8.4 Candidate Host Response Measures and Research Readiness

Candidate host response measures remain investigational. Testicular volume, FSH, and inhibin B were used in one prospective cohort to predict natural‐conception live birth, a couple‐level endpoint [96]; female and couple covariates were not reported in the accessible primary record, so the model should not be presented as a validation of male‐intrinsic reserve. Male AMH [1, 60], seminal total antioxidant capacity [31, 53], HSPA2 [53, 55, 56], hOGG1 genotype [41], and multi‐omic signatures [85, 86, 87] remain research‐only pending assay standardization, independent replication, and incremental predictive value. Several datasets derive from adolescents, and cross‐center comparison requires standardized pre‐analytical handling and platforms. Table 3 maps measures to the evidence source and research readiness without interpreting them as validated reserve markers.

**TABLE 3:** Candidate measures mapped to lesion‐related exposure and damage, proposed host response domains, and couple‐level variables, with the strongest evidence currently available and present research readiness. Entries marked “Research” identify measurements that remain unvalidated. Female age and ovarian reserve are listed separately because pregnancy and live birth are couple‐level outcomes and require explicit couple‐level modeling.

Framework component	Candidate measure	Strongest available evidence	Interpretation and limitations	Current applicability
Lesion‐related exposure	Clinical grade; Doppler reflux	E2—human clinical studies [81]	Imperfect surrogate for cumulative hemodynamic and thermal exposure	Routine characterization
Redox state	Seminal ROS; sORP (MiOXSYS)	Seminal ROS: E2, varicocele‐specific [27, 47]; sORP: E3, assay‐validation evidence from general male‐factor cohorts [92, 93, 94]	Platform‐ and concentration‐dependent; cutoffs not transferable between laboratories	Research protocols only
Molecular damage	Sperm DNA fragmentation	E2—human studies and meta‐analysis [28, 75]	Assay‐specific thresholds; no universally accepted cut‐off	Selected clinical use
Oxidative damage	8‐OHdG; malondialdehyde	E2—human varicocele‐specific 8‐OHdG studies [32, 33]	Downstream damage readout, not a measure of reserve	Research
Antioxidant/redox measures	Seminal TAC; SOD, GPx, GSH/NRF2 pathways	E2 for varicocele biomarker/proteomic associations and E3 for general human redox evidence; E5 for any reserve interpretation [31, 53]	State‐dependent; higher values may accompany different redox states, so direction and functional form require prospective testing	Exploratory nonlinear predictor; no validated score
DNA‐damage processing	hOGG1 genotype and function	E2—single human varicocele cohort [41]	Candidate hOGG1‐associated correlate; not independently replicated	Research
Sperm epigenetic state	Global sperm DNA methylation; 5‐hmC	E2—human varicocele cohorts [42, 43]; E4 for the TET2 mechanism, animal [44]	Grade‐dependent change reported; no reserve interpretation and no outcome data	Research
Apoptosis‐related measures	Bcl‐2/Bax balance; caspase activity	E4—animal models [22]; human expression data descriptive [45, 46]	Candidate mechanistic correlate in animal models; no evidence of a human individual threshold and no validated living‐man assay	No validated assay
Heat‐stress response	HSPA2	E2—human cross‐sectional and proteomic studies [53, 55, 56]	Exploratory marker; cross‐sectional data in adolescent and adult cohorts; no prospective fertility validation	Research
Testicular functional capacity	Testicular volume; FSH; inhibin B	E1—human prognostic models [96]	Male‐variable model for a couple live‐birth endpoint; female/couple covariate handling not verifiable; prognostic association is not proof of reserve	Emerging prediction
Leydig‐cell function	Testosterone; postoperative 17‐hydroxyprogesterone response	E2—human studies [34, 59]	Testosterone is clinically available; postoperative 17‐OHP remains investigational and cannot validate pre‐outcome reserve	Clinical/research
Sertoli‐cell function	Male AMH	E2 for the varicocele endocrine data, E3 for male AMH generally—inconsistent human evidence [1, 60]	Age‐ and assay‐dependent; no uniform hormonal signature and no validated reserve interpretation	Research
Couple‐level modification	Female age; ovarian reserve	E1 for assisted‐reproduction outcomes after varicocelectomy, E3 for the couple‐level DNA‐damage data—guideline and couple‐level evidence [68, 69]	Couple‐level prognostic variables; do not demonstrate oocyte‐mediated repair or male reserve	Routine couple assessment

Abbreviations: 5‐hmC, 5‐hydroxymethylcytosine; 8‐OHdG, 8‐hydroxy‐2′‐deoxyguanosine; AMH, anti‐Müllerian hormone; Bcl‐2, B‐cell lymphoma 2; DFI, DNA fragmentation index; FSH, follicle‐stimulating hormone; GPx, glutathione peroxidase; GSH, glutathione; hOGG1, human 8‐oxoguanine DNA glycosylase 1; HSPA2, heat‐shock protein family A member 2; NRF2, nuclear factor erythroid 2–related factor 2; ROS, reactive oxygen species; SDF, sperm DNA fragmentation; SOD, superoxide dismutase; sORP, static oxidation–reduction potential; TAC, total antioxidant capacity; TET2, ten‐eleven translocation methylcytosine dioxygenase 2; 17‐OHP, 17‐hydroxyprogesterone.

## 9 Clinical Boundary Conditions and Research Translation

Current guidance is summarized only to define the boundary between established practice and the research questions generated by the framework. The framework neither reinterprets guideline recommendations as evidence for itself nor adds a treatment indication. No investigational marker discussed below should influence whether or when repair is offered outside a validated protocol.

### 9.1 Guideline Synthesis and Its Stratification Logic

Formal guidance conditions treatment on a specific conjunction of findings rather than on the lesion alone. The AUA/ASRM guideline addresses varicocele repair in infertile couples when the male partner has a palpable lesion and abnormal semen parameters [5, 97, 98]. This is a clinical decision rule, not evidence of a biological threshold or an unmeasured recoverable‐reserve term. The supporting randomized trial was restricted to couples in whom female infertility was excluded and the woman was younger than 35 years [98]. Its pregnancy result is a couple‐level treatment effect and does not isolate a male reserve mechanism. The research question is whether outcome‐independent host response measures add prediction to established clinical inputs.

The European Association of Urology similarly frames evaluation around identifying and treating modifiable risk factors, positions sperm DNA fragmentation as an emerging biomarker, and cautions against the widespread empirical use of unvalidated treatments and interventions [95]. That this logic is unevenly applied is documented: a large global practice survey found wide disagreement across most aspects of varicocele management, and situations with no guideline recommendation [99]. More recent guidance reinforces this stratified, evidence‐graded approach: the 2025 World Health Organization infertility guideline issued only conditional recommendations on varicocele and made no recommendation for or against antioxidant supplementation in men, reflecting predominantly low‐certainty evidence [100], while the Global Andrology Forum clinical guidelines on the management of infertile men with varicocele [101] provide a dedicated, andrology‐specific framework for patient selection. Although these guidelines stratify care, that structure should not be interpreted as evidence for a biological threshold; none recommends the proposed reserve‐related markers for routine use.

Contemporary guidance combines anatomical, semen, and couple‐level information rather than anatomy alone. Although this stratifies care, it should not be interpreted as evidence for a biological threshold. No guideline currently recommends male AMH, inhibin B, seminal total antioxidant capacity, HSPA2, or repair‐gene testing as a routine determinant of varicocele treatment. These measures remain candidates for research validation, not components of current clinical decision‐making.

### 9.2 Predictors of Response to Varicocelectomy

Baseline semen parameters, testicular volume, vein diameter, endocrine variables, and sperm DNA damage are prognostic or response correlates, not measures locating a man relative to a threshold. The Samplaski and Jang [102, 103] models predict post‐repair male semen parameters, while the study in non‐obstructive azoospermia concerns sperm retrieval or return to the ejaculate [104]. None predicts pregnancy or live birth. Systematic appraisals have also identified baseline semen, Doppler, sperm DNA fragmentation, and laterality variables associated with the male response [90, 105]; these remain exposure, damage, or function descriptors rather than independent host response measures. A separate prospective cohort predicted couple live birth from male testicular volume, FSH, and inhibin B [96], but female/couple covariate handling could not be verified. These studies demonstrate prognostic modeling feasibility without validating a reserve construct.

### 9.3 Research Pathway Toward Prediction With Candidate Measures

Varicocele‐specific prediction models exist for postoperative semen parameters, and one has undergone external multicenter evaluation, although its explanatory performance was lower than in the development cohort (Pearson *R* = 0.328 versus *R* = 0.581) [102, 103]. In one study, the postoperative change in 17‐hydroxyprogesterone was associated with total motile count change in hypogonadal men, whereas eugonadal men showed no comparable shift [59]; this is an outcome‐correlated response measure. A prospective model incorporating testicular volume, FSH, and inhibin B predicted natural‐conception live birth after microsurgical varicocelectomy [96]. These studies show that multivariable prediction is feasible but do not validate the threshold‐resilience construct: most predict a postoperative semen outcome rather than an interaction between measured exposure and a prespecified pre‐outcome host response measure, and external validation remains incomplete. The appropriate next step is to test whether candidate measures add reproducible predictive value to existing clinical models.

### 9.4 Practical Decision Points and Unresolved Trade‐Offs

Translated to the bedside, the framework does not generate a new treatment algorithm. Clinical management should remain guideline based, and the material below is separated into what current evidence supports in practice and what would need to be measured in research protocols before any host response‐informed approach could be evaluated. No element of current care is conditioned on a marker that this review characterizes as inconsistent or research‐grade.

#### 9.4.1 Current Guideline‐Based Care

Varicocele repair may be considered when the male partner is attempting conception and has a palpable clinical varicocele, documented couple infertility, and abnormal semen parameters, after evaluation of the couple and exclusion of major alternative causes [5, 97, 98]. Subclinical varicocele, or a normal semen analysis alone, is not an indication for routine repair on fertility grounds [95, 97]. Sperm DNA fragmentation may inform shared decision‐making in selected men with otherwise unexplained infertility, recurrent pregnancy loss, or assisted‐reproduction failure, but assay limitations and the influence of female age, ovarian reserve, and treatment delay must be weighed [28, 95, 101, 106]. The expected postoperative latency—three to six months before a plateau—should be balanced against the couple's available reproductive time and assisted‐reproduction options [78].

#### 9.4.2 Research‐Only Overlay

Within prospective studies, the same patients may undergo prespecified measurement of testicular volume, FSH, inhibin B, seminal reactive oxygen species, and oxidation‐reduction potential, total antioxidant capacity, HSPA2, hOGG1 genotype, and omics‐derived markers [1, 30, 41, 60, 96]. These measurements should not be used to recommend, withhold, or time repair outside a validated protocol; their purpose is to test incremental prediction and exposure‐by‐prespecified‐measure interactions, not to create a bedside algorithm.

Over‐ and undertreatment are general clinical risks; current evidence does not show that they arise from an unmeasured reserve axis or permit classification relative to a threshold. The documented gaps are assay standardization, validation of candidate host response measures, and separation of the male laboratory response from couple reproductive outcomes. Table 4 distinguishes guideline‐supported inputs from variables that remain investigational.

**TABLE 4:** Current clinical inputs versus investigational variables. Variables are grouped by how far the evidence currently supports their use, not by their place in the model. No variable below the “Selected adjunct” tier should determine whether or when varicocele repair is offered; the research‐only tiers state the evidence future host response research would require.

Tier	Variables	What it contributes	Recommended use
Current clinical inputs—male	Palpable (clinical) varicocele; semen analysis and total motile sperm count; testicular volume; infertility duration [5, 81, 90]	Documents lesion and current male phenotype; residual output may be prognostic, but these inputs do not establish mechanism or reserve	Routine; guideline‐concordant
Current clinical inputs—couple	Documented couple infertility; female age; ovarian reserve [5, 68, 69, 95]	Establishes reproductive context and is associated with pregnancy outcome within the time available; it does not demonstrate repair‐mediated buffering of a male phenotype	Routine; required before attributing subfertility to the varicocele
Selected adjunct	Sperm DNA fragmentation [28, 75]	Molecular damage readout closer to the mechanism than conventional semen analysis	Selected cases, within shared decision‐making; assay‐dependent
Emerging prognostic variables	FSH; inhibin B [60, 96]	Indirect readouts of Sertoli and feedback functional capacity; incorporated into a prospective live‐birth model	Emerging; findings in varicocele remain inconsistent (Table 2) and not a determinant of treatment
Research‐only—redox	Seminal ROS and ORP; total antioxidant capacity [30, 31, 92]	Redox state and antioxidant defense; TAC may be non‐monotonic (Table 3)	Research protocols only
Research‐only—molecular	HSPA2; hOGG1 genotype; male AMH [1, 41, 55, 56, 60]	Candidate heat‐response, hOGG1‐associated, and Sertoli correlates	Research protocols only
Research‐only — omics	miRNA, proteomic, and metabolomic signatures [85, 86, 87, 107, 108, 109]	Proposed composite host response indices; no index has been validated	Research protocols only

Abbreviations: AMH, anti‐Müllerian hormone; FSH, follicle‐stimulating hormone; hOGG1, human 8‐oxoguanine DNA glycosylase 1; HSPA2, heat‐shock protein family A member 2; miRNA, microRNA; ORP, oxidation–reduction potential; ROS, reactive oxygen species; TAC, total antioxidant capacity.

## 10 Knowledge Gaps and Future Research Directions

Stating the framework explicitly exposes which proposed domains can be measured and which remain latent labels. The priorities below are selected for their ability to test whether independently measured host response variables add reproducible information beyond exposure, damage, conventional semen, and couple‐level factors. They do not assume that a reserve axis or threshold exists. Proposed studies are research directions rather than evidence; citations document the current state of the field.

### 10.1 Absence of a Validated, Operationalized Threshold Model

The fundamental gap is not the absence of prediction models. Existing nomograms predict postoperative semen parameters, and one prospective model predicts a couple live‐birth endpoint from male testicular volume, FSH, and inhibin B [96]. What is missing is a prospective test that prespecifies independent measures of lesion‐related exposure, downstream damage, candidate host response variables, and male and couple‐level outcomes. Competing monotonic, nonlinear, and change‐point models should be compared, and any estimated change point must be externally replicated. Until then, neither an independent reserve construct nor a critical threshold has been operationalized.

### 10.2 Under‐Studied Candidate Host Response Measures

Candidate host response measures are under‐validated. AMH and inhibin B findings in a varicocele are inconsistent, and their regulation by FSH and intratesticular testosterone complicates interpretation [1, 60]. Standardized studies should measure these variables before outcomes and test their added predictive value rather than label them Sertoli reserve. Intratesticular testosterone is not directly measured in the reviewed clinical studies; postoperative 17‐OHP is an outcome‐correlated response signal, not a preoperative reserve measure [59]. Seminal TAC and hOGG1‐related measures likewise require independent validation [31, 41]. Oocyte‐mediated repair of varicocele‐associated sperm damage has not been demonstrated in humans and remains a separate couple‐level research hypothesis.

### 10.3 Need for Dedicated Longitudinal Dyadic Cohorts

The affirmative core—that independently measured male host response variables predict a preserved male phenotype and a couple reproductive outcome—was not tested directly in the evidence identified. Existing datasets represent male partners of achieved pregnancies, prior paternity, author‐defined fertile or normozoospermic controls, or incidental lesions rather than prospective dyadic cohorts [8, 71, 72, 73, 74]. A decisive study would measure lesion exposure, downstream damage, and prespecified candidate host response measures before outcomes, then follow both male biological trajectories and time‐to‐pregnancy or live birth. Couple analyses must model female age, ovarian reserve, infertility duration, and intercourse or assisted‐reproduction exposure. Reproducible added prediction or interaction would support the hypothesis; the absence of either would favor simpler models.

### 10.4 Multi‐Omics, Composite Indices, and Methodological Standardization

Candidate genetic, epigenetic, redox‐proteomic, and metabolomic variables are currently studied with heterogeneous methods and mainly cross‐sectional designs [37, 41, 42, 107, 108, 109]. No composite host response index has been independently validated. Future multi‐omics work should use prespecified derivation and external‐validation cohorts and compare added prediction with conventional models. Cross‐laboratory harmonization of DFI, ORP, and candidate assays is a prerequisite to generalizable estimates [28, 30, 31]. Table 5 maps these research gaps without presuming a reserve axis or threshold.

**TABLE 5:** Research agenda mapped to the framework's measurement gaps, with the finding that would weaken or refute the framework in each case. Entries are proposals, not evidence; citations document the current state of the literature, not projected results. The final column is stated so that a null result is informative rather than merely inconclusive.

Gap	Current state	Proposed study design	Primary endpoint/output	Finding that would weaken or refute the framework
Unvalidated nonlinear/change‐point hypothesis	No replicated threshold or change point	Cohorts with independently measured exposure, host response variables, and outcomes; spline/change‐point comparison	Test whether a reproducible change point exists; do not assume one	A strictly linear or monotonic fit, with no independently reproducible change point
Weak candidate host response instrumentation	AMH/inhibin B inconsistent [1, 60]; intratesticular T unmeasured; TAC research‐grade [31, 53]	Powered, standardized biomarker‐trajectory studies with outcome‐independent measurement	Validated candidate‐measure panel	Candidate measures show no reproducible association with outcome after exposure and damage are accounted for
No longitudinal dyadic cohort	Cross‐sectional comparator arms only [72]	Prospective dyadic cohort; baseline exposure, damage, candidate host response, and female/couple variables	Male trajectories and time‐to‐pregnancy/live birth; incremental prediction/interaction	No reproducible exposure‐by‐prespecified‐measure interaction at matched exposure
No validated composite host response index	Susceptibility data idiopathic/single‐cohort [37, 41, 42]; no index	Multi‐omics derivation plus external validation; candidate host response prediction model	Calibrated composite association model	No incremental predictive value over a conventional clinical model
Assay non‐standardization	DFI heterogeneity [28, 31]; ORP validation pending [30]	Cross‐laboratory harmonization; reference‐range establishment	Standardized, comparable exposure, damage, and candidate host response assays	Equivalent prediction from grade and baseline semen parameters alone, or failure of external validation

Abbreviations: AMH, anti‐Müllerian hormone; DFI, DNA fragmentation index; ORP, oxidation–reduction potential; TAC, total antioxidant capacity.

### 10.5 Evidence Identified in the August 26, 2026 Update

Two 2026 studies extend molecular phenotyping without resolving causality. In a pilot study of 25 young men with a left clinical varicocele, semen‐defined subgroups showed distinct serum‐metabolomic and gut‐microbial profiles [110]. In 63 infertile men studied before and after a varicocelectomy, outcome‐associated sperm small‐RNA changes accompanied semen improvement [111]. Neither study demonstrates that a prospectively measured host marker preserves fertility or predicts treatment response independently of the baseline phenotype.

Two recent cohorts expanded the prediction of the male semen response. Baseline left‐testicular mean transit time predicted improvement six months after scleroembolization [112], whereas preoperative DFI, abnormal morphology, and progressive motility predicted complete three‐month semen recovery after repair [113]. These models use exposure, damage, or current‐function descriptors and semen endpoints; neither independently measures reserve, reports couple‐level outcomes, or has external validation.

A varicocele‐specific platform study found low agreement across global SDF and double‐strand‐break readouts, reinforcing that the lesion class and assay platform are not interchangeable [114]. In indirect ART cohorts, the cumulative live birth declined with increasing DFI after IVF but not ICSI [115], while paternal age and the DFI were associated with embryo aneuploidy after maternal‐factor adjustment but not with clinical pregnancy after euploid transfer [116]. These studies refine measurement and couple‐level modeling; none validates an independent host response construct, an exposure‐by‐prespecified‐measure interaction, or a threshold/change point.

## 11 Conclusions

Varicocele‐associated male reproductive phenotypes are heterogeneous, but heterogeneity alone does not establish a reserve mechanism. The present contribution is an evidence‐mapped, hypothesis‐generating structure that separates lesion‐related exposure, candidate host response variables, and couple‐level modifiers, and it translates uncertainty into prospectively testable questions and explicit refutation criteria. It is intended primarily for andrology and reproductive biology researchers, while giving clinicians a clear boundary between guideline‐based management and investigational markers.

No new treatment rule follows from the framework. Current care should remain guideline based, and candidate host response measures should not guide treatment outside research protocols. The framework would gain support only if prespecified, independently measured variables add reproducible predictive value beyond anatomy, semen analysis, redox burden, damage measures, and couple‐level factors in external validation. Failure to do so would favor simpler explanations. Until then, the framework is a research agenda rather than an explanatory mechanism or clinical algorithm.

## Funding

The authors have nothing to report.

## Disclosure

Figures 1 and 2 were created in BioRender from author‐specified scientific content. The authors reviewed and approved the figures and take full responsibility for their accuracy and final form.

## Ethics Statement

This narrative review did not involve new studies on human participants or animals and therefore did not require ethics committee approval.

Animal Studies: This article does not contain any studies with animal subjects performed by any of the authors.

## Consent

This article does not contain any studies with human participants performed by any of the authors. As a narrative review of previously published literature, it did not require informed consent.

## Conflicts of Interest

The authors declare no conflicts of interest.

## Supporting information


**Table S1:** PubMed search strategy and update‐screening account.
**Table S2:** Unit of analysis, female‐partner handling, and permissible inference in audited primary human outcome studies.
**Table S3:** Claim‐level evidence‐tier distribution and coding rules.

## Data Availability

Data sharing not applicable to this article as no datasets were generated or analysed during the current study.
