# Supplementary material for: Male Reproductive Phenotype Heterogeneity in Varicocele: A Hypothesis‐Generating Threshold‐Resilience Framework and Research Agenda
Source: Reprod Med Biol. 2026 Oct 15;25(1):e70102. doi: 10.1002/rmb2.70102 (PMC13671405; doi:10.1002/rmb2.70102)
Supplement: Supplementary file 1 [file RMB2-25-e70102-s001.docx]

Supplementary Tables S1–S3

**Interpretive boundary:** This is a structured narrative review. The search and study-unit tables document transparency; they do not convert the synthesis into a systematic review or a formal risk-of-bias assessment.

# Supplementary Table S1. PubMed search strategy and update-screening account

PubMed was the sole bibliographic database. All queries were executed on 26 August 2026, without a language filter, for records dated 1 January 2015 through 26 August 2026. The update column isolates records dated 1 July–26 August 2026. Counts overlap across queries and are not a PRISMA flow.

| **Strand** | **Exact PubMed query as entered** | **Executed** | **Full hits** | **Update hits** |
| --- | --- | --- | --- | --- |
| A1_preserved_fertility | (varicocele AND (fertile OR fertility OR normozoosperm* OR "normal semen" OR "proven fertile")) AND ("2015/01/01"[Date - Publication] : "2026/08/26"[Date - Publication]) | 2026/08/26 | 621 | 14 |
| A2_hemodynamic_thermal_hypoxic | (varicocele AND (reflux OR "venous stasis" OR hypoxia OR "hypoxia-inducible factor" OR HIF-1* OR hyperthermia OR "scrotal temperature" OR "counter-current")) AND ("2015/01/01"[Date - Publication] : "2026/08/26"[Date - Publication]) | 2026/08/26 | 172 | 8 |
| A3_inflammatory_microenvironmental | (varicocele AND (inflammat* OR cytokine* OR interleukin* OR "tumor necrosis factor" OR leukocytospermia OR "blood-testis barrier" OR "tight junction")) AND ("2015/01/01"[Date - Publication] : "2026/08/26"[Date - Publication]) | 2026/08/26 | 181 | 3 |
| A4_redox_antioxidant | (varicocele AND ("oxidative stress" OR ROS OR "reactive oxygen species" OR "oxidation-reduction potential" OR ORP OR 8-OHdG OR antioxidant* OR "total antioxidant capacity")) AND ("2015/01/01"[Date - Publication] : "2026/08/26"[Date - Publication]) | 2026/08/26 | 383 | 9 |
| A5_dna_damage_repair | (varicocele AND ("DNA fragmentation" OR "DNA damage" OR "DNA repair" OR OGG1 OR hOGG1 OR "base excision repair")) AND ("2015/01/01"[Date - Publication] : "2026/08/26"[Date - Publication]) | 2026/08/26 | 270 | 9 |
| A6_genetic_epigenetic_apoptotic | (varicocele AND (polymorphism* OR genotype OR methylation OR epigenetic* OR TET2 OR apoptos* OR Bcl-2 OR Bax)) AND ("2015/01/01"[Date - Publication] : "2026/08/26"[Date - Publication]) | 2026/08/26 | 207 | 4 |
| A7_heat_shock_thermotolerance | (varicocele AND (HSPA2 OR HSP90* OR "heat shock" OR "heat-shock protein" OR thermotolerance OR "stress granule")) AND ("2015/01/01"[Date - Publication] : "2026/08/26"[Date - Publication]) | 2026/08/26 | 27 | 0 |
| A8_omics | (varicocele AND (proteomic* OR metabolomic* OR transcriptomic* OR microRNA OR miRNA OR "multi-omics")) AND ("2015/01/01"[Date - Publication] : "2026/08/26"[Date - Publication]) | 2026/08/26 | 75 | 2 |
| A9_endocrine_function | (varicocele AND ("inhibin B" OR "anti-Mullerian hormone" OR AMH OR FSH OR testosterone OR "17-hydroxyprogesterone" OR "testicular volume" OR "Sertoli cell" OR "Leydig cell")) AND ("2015/01/01"[Date - Publication] : "2026/08/26"[Date - Publication]) | 2026/08/26 | 402 | 7 |
| A10_predictors_outcomes_after_repair | (varicocelectomy AND (predict* OR nomogram OR "prediction model" OR pregnancy OR "live birth" OR "treatment response" OR recurrence)) AND ("2015/01/01"[Date - Publication] : "2026/08/26"[Date - Publication]) | 2026/08/26 | 346 | 11 |
| B1_gst_male_infertility | (("glutathione S-transferase" OR GSTM1 OR GSTT1 OR GSTP1) AND ("male infertility" OR "semen quality" OR spermatozoa)) AND ("2015/01/01"[Date - Publication] : "2026/08/26"[Date - Publication]) | 2026/08/26 | 145 | 2 |
| B2_oxidative_stress_male_infertility | (("oxidative stress" OR "reactive oxygen species") AND "male infertility" AND (antioxidant* OR "seminal plasma" OR "lipid peroxidation")) AND ("2015/01/01"[Date - Publication] : "2026/08/26"[Date - Publication]) | 2026/08/26 | 955 | 28 |
| B3_sdf_assays_thresholds | (("sperm DNA fragmentation" OR "DNA fragmentation index" OR SCSA OR TUNEL OR "sperm chromatin dispersion" OR "comet assay") AND (threshold OR cut-off OR standardi* OR reproducibility OR "assay variability")) AND ("2015/01/01"[Date - Publication] : "2026/08/26"[Date - Publication]) | 2026/08/26 | 836 | 34 |
| B4_orp_performance | (("oxidation-reduction potential" OR sORP OR MiOXSYS) AND (male AND (infertility OR semen)) AND ("reference range" OR cut-off OR sensitivity OR specificity OR validation)) AND ("2015/01/01"[Date - Publication] : "2026/08/26"[Date - Publication]) | 2026/08/26 | 35 | 0 |
| B5_amh_inhibin_b | (("anti-Mullerian hormone" OR "inhibin B") AND male AND (infertility OR spermatogenesis OR "Sertoli cell" OR "testicular function")) AND ("2015/01/01"[Date - Publication] : "2026/08/26"[Date - Publication]) | 2026/08/26 | 776 | 15 |
| B6_sperm_lesions_embryo | (("single-strand break*" OR "double-strand break*" OR "late paternal effect") AND (spermatozoa OR sperm) AND (embryo* OR "zygotic genome activation")) AND ("2015/01/01"[Date - Publication] : "2026/08/26"[Date - Publication]) | 2026/08/26 | 45 | 2 |
| C1_oocyte_repair | ((oocyte OR zygote OR embryo) AND ("sperm DNA damage" OR "paternal DNA damage") AND (repair OR "DNA repair" OR "repair capacity")) AND ("2015/01/01"[Date - Publication] : "2026/08/26"[Date - Publication]) | 2026/08/26 | 32 | 0 |
| C2_sdf_art_outcomes | ("sperm DNA fragmentation" AND (IVF OR ICSI OR "assisted reproduction*") AND (implantation OR pregnancy OR "live birth" OR miscarriage)) AND ("2015/01/01"[Date - Publication] : "2026/08/26"[Date - Publication]) | 2026/08/26 | 293 | 9 |
| C3_female_factors_outcomes | (("female age" OR "maternal age" OR "ovarian reserve") AND (infertility OR "assisted reproduction*") AND (pregnancy OR "live birth" OR "oocyte quality")) AND ("2015/01/01"[Date - Publication] : "2026/08/26"[Date - Publication]) | 2026/08/26 | 2802 | 63 |

## S1B. Update-screening account

The 19 update queries produced 220 query-level hits and 172 unique PMIDs. Official PubMed ESearch/ESummary metadata were retrieved for all 172 records. A single reviewer screened the records at record level; 14 were retained for detailed assessment and 158 were excluded under one primary reason. The PMID-level ledger is reported below. The retracted rat-varicocele record (PMID 42260699) was not cited.

| **Primary exclusion reason** | **n** |
| --- | --- |
| Female/ART/perinatal topic outside prespecified couple-level question | 42 |
| Non-varicocele human study lacked claim-level relevance | 23 |
| Nonhuman/in-vitro study did not fill a prespecified E4 mechanistic gap | 30 |
| Retraction | 1 |
| Secondary source added no claim-relevant primary or clinical-boundary evidence | 36 |
| Unrelated keyword collision | 17 |
| Varicocele technical/procedural or otherwise non-germane endpoint | 9 |
| Total excluded | 158 |

Hand-searching: reference lists of retained reports and websites or linked records of AUA/ASRM, EAU, WHO, and the Global Andrology Forum were checked most recently on 26 August 2026. Foundational pre-2015 reports were identified by backward citation searching. The initial iterative search did not retain prospective de-duplicated retrieval totals or a full-text exclusion log and cannot be reconstructed as a systematic-review flow.

## S1C. Record-level update-screening ledger

Decision and disposition are shown for every unique PMID returned by the update queries. The ledger documents the single-reviewer screen.

| **PMID** | **Decision** | **Primary reason** | **Detailed assessment** | **Manuscript disposition** | **Citation** | **Notes** |
| --- | --- | --- | --- | --- | --- | --- |
| 40459248 | exclude | Non-varicocele human study lacked claim-level relevance | no | Not cited |  | Title/record-level screening identified a human male or reproductive study without varicocele and without claim-level relevance to the revised framework. Screened record: "Cryptorchidism: Novel genetic insights into CCDC149 mutations." PubMed types: Journal Article; Case Reports; Research Support, Non-U.S. Gov't; source/date: Andrology, 2026 Jul; query strands: B5_amh_inhibin_b; DOI: 10.1111/andr.70072. |
| 41139020 | exclude | Female/ART/perinatal topic outside prespecified couple-level question | no | Not cited |  | Title/record-level screening identified a female, ART, embryo, pregnancy, or perinatal focus outside the prespecified male-varicocele/couple-level question. Screened record: "Artificial intelligence-driven precision treatment of reproductive medicine-related diseases: the optimal protocol choice for IVF-ET." PubMed types: Journal Article; Research Support, Non-U.S. Gov't; source/date: J Adv Res, 2026 Jul; query strands: C3_female_factors_outcomes; DOI: 10.1016/j.jare.2025.10.040. |
| 41386289 | exclude | Varicocele technical/procedural or otherwise non-germane endpoint | no | Not cited |  | The record addressed a procedural, anatomic, technical, grading, or otherwise nongermane varicocele endpoint and did not inform the revised claims. Screened record: "Microsurgical Varicocelectomy with Spermatic Vein to Superficial Epigastric Vein Bypass in Adolescents." PubMed types: Journal Article; source/date: Eur J Pediatr Surg, 2026 Aug; query strands: A2_hemodynamic_thermal_hypoxic, A9_endocrine_function, A10_predictors_outcomes_after_repair; DOI: 10.1055/a-2770-2866. |
| 41531256 | exclude | Non-varicocele human study lacked claim-level relevance | no | Not cited |  | Title/record-level screening identified a human male or reproductive study without varicocele and without claim-level relevance to the revised framework. Screened record: "High Seminal Fluid Calcium Is Associated With Higher Sperm Concentration in Infertile Men and Men From the General Population." PubMed types: Journal Article; Randomized Controlled Trial; source/date: Andrology, 2026 Jul; query strands: B5_amh_inhibin_b; DOI: 10.1111/andr.70172. |
| 41549455 | retain | Retained for detailed assessment | yes | Cited in the main text as [111] for post-varicocelectomy sperm small-RNA changes. | [111] | Study of 63 infertile men before and after varicocelectomy found sperm small-RNA profile changes accompanying improvement in conventional semen parameters. This is a male post-treatment association, not independent validation of reserve. Screened record: "Small RNA profile alterations as a potential mechanism for sperm quality improvement following varicocelectomy." PubMed types: Journal Article; source/date: Asian J Androl, 2026 Jul 1; query strands: A1_preserved_fertility, A6_genetic_epigenetic_apoptotic, A8_omics, A10_predictors_outcomes_after_repair; DOI: 10.4103/aja202568. |
| 41665881 | exclude | Secondary source added no claim-relevant primary or clinical-boundary evidence | no | Not cited |  | PubMed publication type or title identified a review, synthesis, guideline comparison, or committee opinion; it added no claim-relevant primary or clinical-boundary evidence beyond the retained records. Screened record: "The role of robotic surgery in the treatment of advanced endometriosis: a systematic review and meta-analysis." PubMed types: Journal Article; Systematic Review; Meta-Analysis; source/date: Minerva Obstet Gynecol, 2026 Aug; query strands: C3_female_factors_outcomes; DOI: 10.23736/S2724-606X.25.05814-2. |
| 41736309 | exclude | Nonhuman/in-vitro study did not fill a prespecified E4 mechanistic gap | no | Not cited |  | Title/record-level screening identified a nonhuman, ex vivo, or in-vitro model that did not fill a prespecified E4 mechanistic gap in this narrative update. Screened record: "C-phycocyanin protects against obesity-associated type 2 diabetes-induced male fertility dysfunction via key enzyme regulation." PubMed types: Journal Article; source/date: Asian J Androl, 2026 Jul 1; query strands: B1_gst_male_infertility, B2_oxidative_stress_male_infertility; DOI: 10.4103/aja2025108. |
| 41738326 | exclude | Secondary source added no claim-relevant primary or clinical-boundary evidence | no | Not cited |  | PubMed publication type or title identified a review, synthesis, guideline comparison, or committee opinion; it added no claim-relevant primary or clinical-boundary evidence beyond the retained records. Screened record: "Optimizing outcomes in intrauterine insemination: A narrative synthesis of two decades of clinical research (2000-2024)." PubMed types: Journal Article; Review; source/date: Int J Gynaecol Obstet, 2026 Aug; query strands: B3_sdf_assays_thresholds; DOI: 10.1002/ijgo.70847. |
| 41771339 | exclude | Female/ART/perinatal topic outside prespecified couple-level question | no | Not cited |  | Title/record-level screening identified a female, ART, embryo, pregnancy, or perinatal focus outside the prespecified male-varicocele/couple-level question. Screened record: "Association between serum estradiol level decline in the days preceding ovulatory trigger and assisted reproductive technology outcomes." PubMed types: Journal Article; Multicenter Study; source/date: Fertil Steril, 2026 Jul; query strands: C3_female_factors_outcomes; DOI: 10.1016/j.fertnstert.2026.02.030. |
| 41773541 | exclude | Female/ART/perinatal topic outside prespecified couple-level question | no | Not cited |  | Title/record-level screening identified a female, ART, embryo, pregnancy, or perinatal focus outside the prespecified male-varicocele/couple-level question. Screened record: "Advanced Parental Age and Perinatal Risks in Twins Conceived Through In Vitro Fertilization." PubMed types: Journal Article; Research Support, Non-U.S. Gov't; source/date: Andrology, 2026 Jul; query strands: C3_female_factors_outcomes; DOI: 10.1111/andr.70196. |
| 41779255 | retain | Retained for detailed assessment | yes | Detailed assessment completed; not cited because the study concerns adolescent ultrasound grading and testicular asymmetry rather than fertility, semen, or host-response outcomes. |  | Adolescent varicocele study related maximum vein diameter to testicular asymmetry and catch-up and proposed an exploratory threshold around 3.5 mm. It reported no semen, fertility, or host-response outcome. Screened record: "Ultrasound maximum vein diameter contextualizes testicular asymmetry in adolescent varicocele." PubMed types: Journal Article; source/date: Int Urol Nephrol, 2026 Aug; query strands: A2_hemodynamic_thermal_hypoxic; DOI: 10.1007/s11255-026-05075-7. |
| 41833628 | exclude | Secondary source added no claim-relevant primary or clinical-boundary evidence | no | Not cited |  | PubMed publication type or title identified a review, synthesis, guideline comparison, or committee opinion; it added no claim-relevant primary or clinical-boundary evidence beyond the retained records. Screened record: "Varicocele and low testosterone." PubMed types: Journal Article; Review; source/date: Fertil Steril, 2026 Jul; query strands: A9_endocrine_function, A10_predictors_outcomes_after_repair; DOI: 10.1016/j.fertnstert.2026.03.011. |
| 41861980 | exclude | Female/ART/perinatal topic outside prespecified couple-level question | no | Not cited |  | Title/record-level screening identified a female, ART, embryo, pregnancy, or perinatal focus outside the prespecified male-varicocele/couple-level question. Screened record: "The impact of first birth by cesarean delivery on subsequent reproductive outcomes-a population cohort study." PubMed types: Journal Article; source/date: Am J Obstet Gynecol, 2026 Aug; query strands: C3_female_factors_outcomes; DOI: 10.1016/j.ajog.2026.03.006. |
| 41905684 | exclude | Varicocele technical/procedural or otherwise non-germane endpoint | no | Not cited |  | The record addressed a procedural, anatomic, technical, grading, or otherwise nongermane varicocele endpoint and did not inform the revised claims. Screened record: "Transfemoral versus Transjugular Varicocele Embolization: Does It Affect the Technical Success Rate and Radiation Exposure?" PubMed types: Journal Article; Comparative Study; source/date: J Vasc Interv Radiol, 2026 Jul; query strands: A2_hemodynamic_thermal_hypoxic; DOI: 10.1016/j.jvir.2026.108761. |
| 41921892 | exclude | Secondary source added no claim-relevant primary or clinical-boundary evidence | no | Not cited |  | PubMed publication type or title identified a review, synthesis, guideline comparison, or committee opinion; it added no claim-relevant primary or clinical-boundary evidence beyond the retained records. Screened record: "Varicocelectomy in men with nonobstructive azoospermia." PubMed types: Journal Article; Review; source/date: Fertil Steril, 2026 Jul; query strands: A1_preserved_fertility; DOI: 10.1016/j.fertnstert.2026.03.029. |
| 41923441 | exclude | Secondary source added no claim-relevant primary or clinical-boundary evidence | no | Not cited |  | PubMed publication type or title identified a review, synthesis, guideline comparison, or committee opinion; it added no claim-relevant primary or clinical-boundary evidence beyond the retained records. Screened record: "Male testosterone synthesis disorders: oxidative stress pathways, regulatory mechanisms, potential of antioxidant therapy, and botanical medicine prospects." PubMed types: Journal Article; Review; source/date: Asian J Androl, 2026 Jul 1; query strands: B2_oxidative_stress_male_infertility; DOI: 10.4103/aja2025105. |
| 41941939 | exclude | Secondary source added no claim-relevant primary or clinical-boundary evidence | no | Not cited |  | PubMed publication type or title identified a review, synthesis, guideline comparison, or committee opinion; it added no claim-relevant primary or clinical-boundary evidence beyond the retained records. Screened record: "Oncofertility in the era of targeted therapy: Tyrosine kinase inhibitors, female reproductive toxicity, and pregnancy outcomes." PubMed types: Journal Article; Review; source/date: Reprod Toxicol, 2026 Aug; query strands: C3_female_factors_outcomes; DOI: 10.1016/j.reprotox.2026.109237. |
| 41966417 | exclude | Secondary source added no claim-relevant primary or clinical-boundary evidence | no | Not cited |  | PubMed publication type or title identified a review, synthesis, guideline comparison, or committee opinion; it added no claim-relevant primary or clinical-boundary evidence beyond the retained records. Screened record: "Mechanisms of reproductive toxicity and endocrine disruption of bisphenols and per- and polyfluoroalkyl substances (PFAS): Implications for women's reproductive health." PubMed types: Journal Article; Review; source/date: Reprod Toxicol, 2026 Aug; query strands: C3_female_factors_outcomes; DOI: 10.1016/j.reprotox.2026.109239. |
| 41985734 | exclude | Female/ART/perinatal topic outside prespecified couple-level question | no | Not cited |  | Title/record-level screening identified a female, ART, embryo, pregnancy, or perinatal focus outside the prespecified male-varicocele/couple-level question. Screened record: "Postfertilization telomere marker testing in human embryos: a pilot study of a novel embryo evaluation assay." PubMed types: Journal Article; source/date: Fertil Steril, 2026 Aug; query strands: C3_female_factors_outcomes; DOI: 10.1016/j.fertnstert.2026.04.003. |
| 41990882 | exclude | Varicocele technical/procedural or otherwise non-germane endpoint | no | Not cited |  | The record addressed a procedural, anatomic, technical, grading, or otherwise nongermane varicocele endpoint and did not inform the revised claims. Screened record: "Varicocele and Untreated Hypogonadism Synergistically Increase Prostate Cancer Risk via Testosterone Shunt Physiology and Can Be Ameliorated With Testosterone Therapy." PubMed types: Journal Article; source/date: Urology, 2026 Jul; query strands: A9_endocrine_function; DOI: 10.1016/j.urology.2026.04.016. |
| 42000002 | exclude | Secondary source added no claim-relevant primary or clinical-boundary evidence | no | Not cited |  | PubMed publication type or title identified a review, synthesis, guideline comparison, or committee opinion; it added no claim-relevant primary or clinical-boundary evidence beyond the retained records. Screened record: "Multi-component, multi-target actions of Wuzi Yanzong Pill in male infertility: An evidence map linking clinical signals to mechanistic networks." PubMed types: Journal Article; Review; source/date: J Ethnopharmacol, 2026 Aug 10; query strands: B2_oxidative_stress_male_infertility; DOI: 10.1016/j.jep.2026.121705. |
| 42000055 | exclude | Nonhuman/in-vitro study did not fill a prespecified E4 mechanistic gap | no | Not cited |  | Title/record-level screening identified a nonhuman, ex vivo, or in-vitro model that did not fill a prespecified E4 mechanistic gap in this narrative update. Screened record: "Alpha-ketoglutarate alleviates Cadmium-induced ferroptosis in association with enhanced mitochondrial function in testes." PubMed types: Journal Article; source/date: Reprod Toxicol, 2026 Aug; query strands: B2_oxidative_stress_male_infertility; DOI: 10.1016/j.reprotox.2026.109249. |
| 42011123 | exclude | Non-varicocele human study lacked claim-level relevance | no | Not cited |  | Title/record-level screening identified a human male or reproductive study without varicocele and without claim-level relevance to the revised framework. Screened record: "Computer-Assisted Sperm Analysis (CASA) Versus Manual Evaluation and the Clinical Significance of Sperm Hyperactivated Motility." PubMed types: Journal Article; Comparative Study; Research Support, Non-U.S. Gov't; source/date: Andrology, 2026 Jul; query strands: C3_female_factors_outcomes; DOI: 10.1111/andr.70242. |
| 42025539 | exclude | Varicocele technical/procedural or otherwise non-germane endpoint | no | Not cited |  | The record addressed a procedural, anatomic, technical, grading, or otherwise nongermane varicocele endpoint and did not inform the revised claims. Screened record: "Indocyanine Green Fluorescence Lymphography for Lymphatic-Sparing Laparoscopic Palomo Varicocelectomy in Children: 5-Year Experience and Technical Considerations." PubMed types: Journal Article; source/date: J Laparoendosc Adv Surg Tech A, 2026 Jul; query strands: A10_predictors_outcomes_after_repair; DOI: 10.1177/10926429261435770. |
| 42025703 | exclude | Nonhuman/in-vitro study did not fill a prespecified E4 mechanistic gap | no | Not cited |  | Title/record-level screening identified a nonhuman, ex vivo, or in-vitro model that did not fill a prespecified E4 mechanistic gap in this narrative update. Screened record: "GLUL deficiency causes sperm acrosome defects and male infertility via influencing redox balance in mice." PubMed types: Journal Article; source/date: Free Radic Biol Med, 2026 Aug 1; query strands: B2_oxidative_stress_male_infertility; DOI: 10.1016/j.freeradbiomed.2026.04.030. |
| 42049202 | exclude | Secondary source added no claim-relevant primary or clinical-boundary evidence | no | Not cited |  | PubMed publication type or title identified a review, synthesis, guideline comparison, or committee opinion; it added no claim-relevant primary or clinical-boundary evidence beyond the retained records. Screened record: "Sperm DNA fragmentation: how to test, when to test, and what to do with abnormal results-a pragmatic mini-review for clinical practice." PubMed types: Journal Article; Review; source/date: Hum Reprod, 2026 Jul 1; query strands: A1_preserved_fertility, A4_redox_antioxidant, A5_dna_damage_repair, B6_sperm_lesions_embryo, C2_sdf_art_outcomes; DOI: 10.1093/humrep/deag056. |
| 42053703 | retain | Retained for detailed assessment | yes | Cited in the main text as [112] for retrospective prediction of semen response after scleroembolization. | [112] | Retrospective cohort of 85 patients treated by scleroembolization; baseline left testicular mean transit time predicted doubling of total sperm count or total motile sperm count at 6 months. Endpoint was semen response, without a couple outcome or external validation. Screened record: "Predictive parameters of semen outcome improvement after varicocele treatment by scleroembolization: a retrospective study." PubMed types: Journal Article; source/date: J Endocrinol Invest, 2026 Jul; query strands: A1_preserved_fertility; DOI: 10.1007/s40618-026-02844-0. |
| 42066978 | exclude | Secondary source added no claim-relevant primary or clinical-boundary evidence | no | Not cited |  | PubMed publication type or title identified a review, synthesis, guideline comparison, or committee opinion; it added no claim-relevant primary or clinical-boundary evidence beyond the retained records. Screened record: "Adolescent varicocele: a surgical conundrum." PubMed types: Journal Article; Review; source/date: Fertil Steril, 2026 Jul; query strands: A1_preserved_fertility, A2_hemodynamic_thermal_hypoxic, A4_redox_antioxidant, A6_genetic_epigenetic_apoptotic, A9_endocrine_function, A10_predictors_outcomes_after_repair; DOI: 10.1016/j.fertnstert.2026.04.015. |
| 42091361 | exclude | Unrelated keyword collision | no | Not cited |  | The record was retrieved through broad oxidative-stress, repair, or reproduction terms, but title/record-level screening showed an unrelated clinical, organ-system, or laboratory topic. Screened record: "Non-invasive Ultrasound Liver Ablation Using Histotripsy: A Feasibility Study in Ex Vivo Porcine Liver and a Chronic Study in Rodent Models In Vivo." PubMed types: Journal Article; source/date: Ultrasound Med Biol, 2026 Aug; query strands: B3_sdf_assays_thresholds; DOI: 10.1016/j.ultrasmedbio.2026.03.001. |
| 42091403 | exclude | Secondary source added no claim-relevant primary or clinical-boundary evidence | no | Not cited |  | PubMed publication type or title identified a review, synthesis, guideline comparison, or committee opinion; it added no claim-relevant primary or clinical-boundary evidence beyond the retained records. Screened record: "Intracytoplasmic sperm injection for nonmale factor indications: a Committee opinion." PubMed types: Journal Article; Review; source/date: Fertil Steril, 2026 Jul; query strands: C3_female_factors_outcomes; DOI: 10.1016/j.fertnstert.2026.03.032. |
| 42105942 | exclude | Non-varicocele human study lacked claim-level relevance | no | Not cited |  | Title/record-level screening identified a human male or reproductive study without varicocele and without claim-level relevance to the revised framework. Screened record: "Biological variation of serum Anti-Müllerian Hormone in healthy adult men." PubMed types: Journal Article; source/date: Clin Chim Acta, 2026 Aug 15; query strands: B5_amh_inhibin_b; DOI: 10.1016/j.cca.2026.121068. |
| 42128196 | exclude | Secondary source added no claim-relevant primary or clinical-boundary evidence | no | Not cited |  | PubMed publication type or title identified a review, synthesis, guideline comparison, or committee opinion; it added no claim-relevant primary or clinical-boundary evidence beyond the retained records. Screened record: "Varicocele: new perspectives on an old problem." PubMed types: Journal Article; Review; source/date: Fertil Steril, 2026 Jul; query strands: A1_preserved_fertility, A9_endocrine_function; DOI: 10.1016/j.fertnstert.2026.05.147. |
| 42150354 | exclude | Secondary source added no claim-relevant primary or clinical-boundary evidence | no | Not cited |  | PubMed publication type or title identified a review, synthesis, guideline comparison, or committee opinion; it added no claim-relevant primary or clinical-boundary evidence beyond the retained records. Screened record: "Resveratrol supplementation and assisted reproduction outcomes: A systematic review and meta-analysis." PubMed types: Journal Article; Meta-Analysis; Systematic Review; source/date: Eur J Obstet Gynecol Reprod Biol, 2026 Jul; query strands: C3_female_factors_outcomes; DOI: 10.1016/j.ejogrb.2026.115193. |
| 42155227 | exclude | Non-varicocele human study lacked claim-level relevance | no | Not cited |  | Title/record-level screening identified a human male or reproductive study without varicocele and without claim-level relevance to the revised framework. Screened record: "Use of ejaculated versus testicular sperm for assisted reproduction outcomes in patients with oligozoospermia in the absence of sperm DNA fragmentation testing." PubMed types: Journal Article; Randomized Controlled Trial; Clinical Trial, Phase II; Comparative Study; source/date: Eur J Obstet Gynecol Reprod Biol, 2026 Jul; query strands: C2_sdf_art_outcomes; DOI: 10.1016/j.ejogrb.2026.115195. |
| 42155917 | exclude | Secondary source added no claim-relevant primary or clinical-boundary evidence | no | Not cited |  | PubMed publication type or title identified a review, synthesis, guideline comparison, or committee opinion; it added no claim-relevant primary or clinical-boundary evidence beyond the retained records. Screened record: "Can a focus on intracellular signaling assist us with ovarian stimulation in poor-prognosis patients?" PubMed types: Journal Article; Review; source/date: Fertil Steril, 2026 Aug; query strands: C3_female_factors_outcomes; DOI: 10.1016/j.fertnstert.2026.05.149. |
| 42168604 | exclude | Nonhuman/in-vitro study did not fill a prespecified E4 mechanistic gap | no | Not cited |  | Title/record-level screening identified a nonhuman, ex vivo, or in-vitro model that did not fill a prespecified E4 mechanistic gap in this narrative update. Screened record: "20S proteasome-regulated proteostasis in ELVAs is critical for oocyte-to-embryo transition and female fertility." PubMed types: Journal Article; source/date: EMBO J, 2026 Jul; query strands: C3_female_factors_outcomes; DOI: 10.1038/s44318-026-00813-0. |
| 42172876 | exclude | Secondary source added no claim-relevant primary or clinical-boundary evidence | no | Not cited |  | PubMed publication type or title identified a review, synthesis, guideline comparison, or committee opinion; it added no claim-relevant primary or clinical-boundary evidence beyond the retained records. Screened record: "Diabetes-driven oxidative stress and calcium dysregulation in gametogenesis and early fertilization." PubMed types: Journal Article; Review; source/date: J Diabetes Complications, 2026 Jul; query strands: B2_oxidative_stress_male_infertility; DOI: 10.1016/j.jdiacomp.2026.109345. |
| 42184598 | exclude | Nonhuman/in-vitro study did not fill a prespecified E4 mechanistic gap | no | Not cited |  | Title/record-level screening identified a nonhuman, ex vivo, or in-vitro model that did not fill a prespecified E4 mechanistic gap in this narrative update. Screened record: "Protective effects of naringin against heat stress-induced oxidative and reproductive dysfunction in male rats." PubMed types: Journal Article; source/date: J Therm Biol, 2026 Jul; query strands: B2_oxidative_stress_male_infertility; DOI: 10.1016/j.jtherbio.2026.104481. |
| 42191021 | exclude | Unrelated keyword collision | no | Not cited |  | The record was retrieved through broad oxidative-stress, repair, or reproduction terms, but title/record-level screening showed an unrelated clinical, organ-system, or laboratory topic. Screened record: "Deep Learning-Based Positive Region Segmentation and Spatial Registration of Virtual Multiplex Immunohistochemical Whole-Slide Images." PubMed types: Journal Article; source/date: Lab Invest, 2026 Aug; query strands: B3_sdf_assays_thresholds; DOI: 10.1016/j.labinv.2026.106139. |
| 42200911 | exclude | Nonhuman/in-vitro study did not fill a prespecified E4 mechanistic gap | no | Not cited |  | Title/record-level screening identified a nonhuman, ex vivo, or in-vitro model that did not fill a prespecified E4 mechanistic gap in this narrative update. Screened record: "Protective efficacy of Met-Ser-Arg against irradiation-related testicular injury†." PubMed types: Journal Article; source/date: Biol Reprod, 2026 Aug 18; query strands: B2_oxidative_stress_male_infertility; DOI: 10.1093/biolre/ioag110. |
| 42213867 | exclude | Female/ART/perinatal topic outside prespecified couple-level question | no | Not cited |  | Title/record-level screening identified a female, ART, embryo, pregnancy, or perinatal focus outside the prespecified male-varicocele/couple-level question. Screened record: "Cleavage stage versus blastocyst stage transfers in patients with a single zygote: an emulated target trial." PubMed types: Journal Article; source/date: Hum Reprod, 2026 Jul 1; query strands: C3_female_factors_outcomes; DOI: 10.1093/humrep/deag075. |
| 42223195 | exclude | Female/ART/perinatal topic outside prespecified couple-level question | no | Not cited |  | Title/record-level screening identified a female, ART, embryo, pregnancy, or perinatal focus outside the prespecified male-varicocele/couple-level question. Screened record: "Risk of minor congenital heart defect in infants conceived via assisted reproductive technology: cohort study from the Copenhagen Baby Heart Study." PubMed types: Journal Article; Multicenter Study; source/date: Ultrasound Obstet Gynecol, 2026 Jul; query strands: C3_female_factors_outcomes; DOI: 10.1002/uog.70246. |
| 42240942 | exclude | Nonhuman/in-vitro study did not fill a prespecified E4 mechanistic gap | no | Not cited |  | Title/record-level screening identified a nonhuman, ex vivo, or in-vitro model that did not fill a prespecified E4 mechanistic gap in this narrative update. Screened record: "The Protective Effect of Galangin on Histopathological Lesions, Immunohistochemical Findings and Spermatological Parameters in Phthalate-Induced Testicular Damage in Rats." PubMed types: Journal Article; source/date: Anat Histol Embryol, 2026 Jul; query strands: B1_gst_male_infertility; DOI: 10.1111/ahe.70136. |
| 42251806 | exclude | Female/ART/perinatal topic outside prespecified couple-level question | no | Not cited |  | Title/record-level screening identified a female, ART, embryo, pregnancy, or perinatal focus outside the prespecified male-varicocele/couple-level question. Screened record: "Anthropometric outcomes at 12 years after fresh versus frozen embryo transfer compared with natural conception." PubMed types: Journal Article; Multicenter Study; Comparative Study; source/date: Eur J Obstet Gynecol Reprod Biol, 2026 Aug; query strands: C3_female_factors_outcomes; DOI: 10.1016/j.ejogrb.2026.115227. |
| 42260699 | exclude | Retraction | no | Not cited |  | PubMed identifies this record as a Retraction Notice; it was excluded and is not cited. Screened record: "RETRACTION: Melatonin Epigenetic Potential on Testicular Functions and Fertility Profile in Varicocele Rat Model is Mediated by Silent Information Regulator 1." PubMed types: Journal Article; Retraction Notice; source/date: Br J Pharmacol, 2026 Aug; query strands: A1_preserved_fertility, A6_genetic_epigenetic_apoptotic; DOI: 10.1111/bph.70551. |
| 42267954 | exclude | Non-varicocele human study lacked claim-level relevance | no | Not cited |  | Title/record-level screening identified a human male or reproductive study without varicocele and without claim-level relevance to the revised framework. Screened record: "Clinical Use of Inhibin B in Male Adolescent Childhood Cancer Survivors: Correlation With Cyclophosphamide Equivalent Dosing." PubMed types: Journal Article; Observational Study; source/date: J Pediatr Hematol Oncol, 2026 Aug 1; query strands: B5_amh_inhibin_b; DOI: 10.1097/MPH.0000000000003226. |
| 42312322 | exclude | Non-varicocele human study lacked claim-level relevance | no | Not cited |  | Title/record-level screening identified a human male or reproductive study without varicocele and without claim-level relevance to the revised framework. Screened record: "Preserved Spermatogenesis and Hormonal Function in Men Receiving a Pretomanid Regimen (BPaMZ) for Pulmonary Drug-Resistant Tuberculosis." PubMed types: Journal Article; Clinical Trial, Phase II; Multicenter Study; Research Support, N.I.H., Extramural; source/date: Clin Pharmacol Ther, 2026 Aug; query strands: B5_amh_inhibin_b; DOI: 10.1002/cpt.70363. |
| 42314538 | exclude | Non-varicocele human study lacked claim-level relevance | no | Not cited |  | Title/record-level screening identified a human male or reproductive study without varicocele and without claim-level relevance to the revised framework. Screened record: "Ultrasound localisation microscopy tracks testicular microvascular adaptations to endocrine function in male infertility." PubMed types: Journal Article; source/date: EBioMedicine, 2026 Jul; query strands: B5_amh_inhibin_b; DOI: 10.1016/j.ebiom.2026.106333. |
| 42331050 | exclude | Secondary source added no claim-relevant primary or clinical-boundary evidence | no | Not cited |  | PubMed publication type or title identified a review, synthesis, guideline comparison, or committee opinion; it added no claim-relevant primary or clinical-boundary evidence beyond the retained records. Screened record: "Possible therapeutic options for enhancing the number of oocytes in poor ovarian responders." PubMed types: Journal Article; Review; source/date: Fertil Steril, 2026 Aug; query strands: C3_female_factors_outcomes; DOI: 10.1016/j.fertnstert.2026.06.025. |
| 42333713 | exclude | Unrelated keyword collision | no | Not cited |  | The record was retrieved through broad oxidative-stress, repair, or reproduction terms, but title/record-level screening showed an unrelated clinical, organ-system, or laboratory topic. Screened record: "Correlates of MAPK/Nrf2/STAT-3 Pathway Protein Expression With Morin Treatment in Helicobacter pylori-Infected Gastric Epithelial Cells-1 Cells: An Exploratory In Vitro Study." PubMed types: Journal Article; source/date: J Biochem Mol Toxicol, 2026 Jul; query strands: B3_sdf_assays_thresholds; DOI: 10.1002/jbt.70935. |
| 42358470 | exclude | Varicocele technical/procedural or otherwise non-germane endpoint | no | Not cited |  | The record addressed a procedural, anatomic, technical, grading, or otherwise nongermane varicocele endpoint and did not inform the revised claims. Screened record: "Varicocelectomy Restores Spermatogenesis in Fontan-Associated Varicocele: A Two-Case Series." PubMed types: Journal Article; source/date: IJU Case Rep, 2026 Jul; query strands: A10_predictors_outcomes_after_repair; DOI: 10.1002/iju5.70206. |
| 42362260 | exclude | Female/ART/perinatal topic outside prespecified couple-level question | no | Not cited |  | Title/record-level screening identified a female, ART, embryo, pregnancy, or perinatal focus outside the prespecified male-varicocele/couple-level question. Screened record: "The effectiveness of vitrified zygote banking for managing the severe diminished ovarian reserve in infertile women: A retrospective cohort study." PubMed types: Journal Article; source/date: Taiwan J Obstet Gynecol, 2026 Jul; query strands: C3_female_factors_outcomes; DOI: 10.1016/j.tjog.2025.06.010. |
| 42385478 | exclude | Secondary source added no claim-relevant primary or clinical-boundary evidence | no | Not cited |  | PubMed publication type or title identified a review, synthesis, guideline comparison, or committee opinion; it added no claim-relevant primary or clinical-boundary evidence beyond the retained records. Screened record: "Advancing paternal age: Effects on sperm quality and offspring health." PubMed types: Journal Article; Review; source/date: Maturitas, 2026 Aug; query strands: C2_sdf_art_outcomes; DOI: 10.1016/j.maturitas.2026.109042. |
| 42393810 | exclude | Nonhuman/in-vitro study did not fill a prespecified E4 mechanistic gap | no | Not cited |  | Title/record-level screening identified a nonhuman, ex vivo, or in-vitro model that did not fill a prespecified E4 mechanistic gap in this narrative update. Screened record: "Adipose-derived stem cells-conditioned medium (ADMSCs-CM) improves testicular function and sperm DNA damage via Nrf2 antioxidant signaling pathways in rat unilateral varicocele." PubMed types: Journal Article; source/date: Stem Cell Res Ther, 2026 Jul 1; query strands: A4_redox_antioxidant, A5_dna_damage_repair; DOI: 10.1186/s13287-026-05145-8. |
| 42394600 | exclude | Nonhuman/in-vitro study did not fill a prespecified E4 mechanistic gap | no | Not cited |  | Title/record-level screening identified a nonhuman, ex vivo, or in-vitro model that did not fill a prespecified E4 mechanistic gap in this narrative update. Screened record: "Inhalation exposure to heat-not-burn tobacco aerosol versus combustible cigarette smoke: comparative assessment of respiratory and systemic toxicity in an experimental rat model." PubMed types: Journal Article; Comparative Study; source/date: Inhal Toxicol, 2026 Sep; query strands: B3_sdf_assays_thresholds; DOI: 10.1080/08958378.2026.2686703. |
| 42399977 | exclude | Non-varicocele human study lacked claim-level relevance | no | Not cited |  | Title/record-level screening identified a human male or reproductive study without varicocele and without claim-level relevance to the revised framework. Screened record: "Moderate-intensity physical activity is associated with favorable semen-parameter changes and normalization of selected abnormalities in men with impaired semen quality." PubMed types: Journal Article; source/date: Reprod Biol Endocrinol, 2026 Jul 3; query strands: B3_sdf_assays_thresholds; DOI: 10.1186/s12958-026-01585-2. |
| 42400924 | exclude | Non-varicocele human study lacked claim-level relevance | no | Not cited |  | Title/record-level screening identified a human male or reproductive study without varicocele and without claim-level relevance to the revised framework. Screened record: "Pediatric andrology: bridging pediatric urology and reproductive medicine." PubMed types: Journal Article; source/date: Curr Opin Urol, 2026 Jul 2; query strands: A1_preserved_fertility; DOI: 10.1097/MOU.0000000000001422. |
| 42409747 | exclude | Female/ART/perinatal topic outside prespecified couple-level question | no | Not cited |  | Title/record-level screening identified a female, ART, embryo, pregnancy, or perinatal focus outside the prespecified male-varicocele/couple-level question. Screened record: "Association of basic fibroblast growth factor and body mass index with oocyte quality and quantity in infertile women with PCOS and diminished ovarian reserve undergoing ICSI." PubMed types: Journal Article; source/date: Clin Exp Reprod Med, 2026 Jul 7; query strands: C3_female_factors_outcomes; DOI: 10.5653/cerm.2025.08865. |
| 42417195 | exclude | Nonhuman/in-vitro study did not fill a prespecified E4 mechanistic gap | no | Not cited |  | Title/record-level screening identified a nonhuman, ex vivo, or in-vitro model that did not fill a prespecified E4 mechanistic gap in this narrative update. Screened record: "Cap 2'-O-methyltransferase CMTR2 regulates male meiosis independent of its methyltransferase activity." PubMed types: Journal Article; source/date: Nucleic Acids Res, 2026 Jul 3; query strands: B6_sperm_lesions_embryo; DOI: 10.1093/nar/gkag699. |
| 42417943 | exclude | Unrelated keyword collision | no | Not cited |  | The record was retrieved through broad oxidative-stress, repair, or reproduction terms, but title/record-level screening showed an unrelated clinical, organ-system, or laboratory topic. Screened record: "Astragaloside IV pretreatment enhances the therapeutic efficacy of bone marrow mesenchymal stem cells in murine fulminant hepatic failure." PubMed types: Journal Article; source/date: Mol Biol Rep, 2026 Jul 8; query strands: B3_sdf_assays_thresholds; DOI: 10.1007/s11033-026-12051-y. |
| 42419555 | exclude | Varicocele technical/procedural or otherwise non-germane endpoint | no | Not cited |  | The record addressed a procedural, anatomic, technical, grading, or otherwise nongermane varicocele endpoint and did not inform the revised claims. Screened record: "Endovascular Embolization of Left-Sided Varicocele: Outcomes by Venographic Anatomy and Coil-Adjunct Strategy." PubMed types: Journal Article; source/date: J Vasc Interv Radiol, 2026 Jul 9; query strands: A2_hemodynamic_thermal_hypoxic, A10_predictors_outcomes_after_repair; DOI: 10.1016/j.jvir.2026.108949. |
| 42424397 | exclude | Nonhuman/in-vitro study did not fill a prespecified E4 mechanistic gap | no | Not cited |  | Title/record-level screening identified a nonhuman, ex vivo, or in-vitro model that did not fill a prespecified E4 mechanistic gap in this narrative update. Screened record: "Antioxidant and Geno-protective Effects of Naturally Occurring Delphinidin Glycosides." PubMed types: Journal Article; source/date: Mutagenesis, 2026 Jul 9; query strands: B3_sdf_assays_thresholds; DOI: 10.1093/mutage/geag027. |
| 42424759 | exclude | Unrelated keyword collision | no | Not cited |  | The record was retrieved through broad oxidative-stress, repair, or reproduction terms, but title/record-level screening showed an unrelated clinical, organ-system, or laboratory topic. Screened record: "Analysis of indocyanine green-guided sentinel lymph node mapping for cervical cancer during pregnancy." PubMed types: Journal Article; Multicenter Study; Observational Study; source/date: Int J Gynecol Cancer, 2026 Aug; query strands: C3_female_factors_outcomes; DOI: 10.1016/j.ijgc.2026.104810. |
| 42435406 | exclude | Nonhuman/in-vitro study did not fill a prespecified E4 mechanistic gap | no | Not cited |  | Title/record-level screening identified a nonhuman, ex vivo, or in-vitro model that did not fill a prespecified E4 mechanistic gap in this narrative update. Screened record: "Chorioamnionitis and glucocorticoids elicit acute adverse effects on the developing ovine fetal ovaries." PubMed types: Journal Article; source/date: Reproduction, 2026 Aug 4; query strands: B3_sdf_assays_thresholds; DOI: 10.1093/reprod/xaag088. |
| 42436421 | exclude | Unrelated keyword collision | no | Not cited |  | The record was retrieved through broad oxidative-stress, repair, or reproduction terms, but title/record-level screening showed an unrelated clinical, organ-system, or laboratory topic. Screened record: "A novel rat model of neurotrophic keratopathy complicated with limbal stem cell deficiency induced by trigeminal ophthalmic branch injury." PubMed types: Journal Article; source/date: BMC Ophthalmol, 2026 Jul 11; query strands: B3_sdf_assays_thresholds; DOI: 10.1186/s12886-026-05116-w. |
| 42437650 | exclude | Unrelated keyword collision | no | Not cited |  | The record was retrieved through broad oxidative-stress, repair, or reproduction terms, but title/record-level screening showed an unrelated clinical, organ-system, or laboratory topic. Screened record: "Multi-omics reveals the mechanism of Rehmannia glutinosa Libosch. Processed with Amomum villosum Lour. in treating blood deficiency syndrome." PubMed types: Journal Article; source/date: J Ethnopharmacol, 2027 Jan 10; query strands: B3_sdf_assays_thresholds; DOI: 10.1016/j.jep.2026.122184. |
| 42441882 | exclude | Secondary source added no claim-relevant primary or clinical-boundary evidence | no | Not cited |  | PubMed publication type or title identified a review, synthesis, guideline comparison, or committee opinion; it added no claim-relevant primary or clinical-boundary evidence beyond the retained records. Screened record: "Endometriosis as a cause of reduced IVF success rates: guilty beyond reasonable doubt?" PubMed types: Journal Article; Review; source/date: JBRA Assist Reprod, 2026 Jul 13; query strands: C3_female_factors_outcomes; DOI: 10.5935/1518-0557.20260026. |
| 42442304 | exclude | Nonhuman/in-vitro study did not fill a prespecified E4 mechanistic gap | no | Not cited |  | Title/record-level screening identified a nonhuman, ex vivo, or in-vitro model that did not fill a prespecified E4 mechanistic gap in this narrative update. Screened record: "Amelioration of obesity-induced testicular dysfunction and structural damage by ganoderma lucidum polysaccharides and triterpenoids in rats: Correlation with Nrf2-mediated antioxidant response." PubMed types: Journal Article; source/date: Biomed Pharmacother, 2026 Aug; query strands: B2_oxidative_stress_male_infertility, B5_amh_inhibin_b; DOI: 10.1016/j.biopha.2026.119765. |
| 42444480 | exclude | Female/ART/perinatal topic outside prespecified couple-level question | no | Not cited |  | Title/record-level screening identified a female, ART, embryo, pregnancy, or perinatal focus outside the prespecified male-varicocele/couple-level question. Screened record: "Effect of hysteroscopic metroplasty on IVF outcomes in women with T-shaped uterus: a retrospective cohort study." PubMed types: Journal Article; source/date: J Obstet Gynaecol, 2026 Dec; query strands: C3_female_factors_outcomes; DOI: 10.1080/01443615.2026.2699350. |
| 42449482 | exclude | Unrelated keyword collision | no | Not cited |  | The record was retrieved through broad oxidative-stress, repair, or reproduction terms, but title/record-level screening showed an unrelated clinical, organ-system, or laboratory topic. Screened record: "Prenatal aerosol exposure and neonatal cord blood hepatic enzyme activities in a highly polluted urban area." PubMed types: Journal Article; source/date: Int J Environ Health Res, 2026 Jul 14; query strands: C3_female_factors_outcomes; DOI: 10.1080/09603123.2026.2701102. |
| 42451718 | exclude | Nonhuman/in-vitro study did not fill a prespecified E4 mechanistic gap | no | Not cited |  | Title/record-level screening identified a nonhuman, ex vivo, or in-vitro model that did not fill a prespecified E4 mechanistic gap in this narrative update. Screened record: "Subcritical Water Extraction Enables the Production of Cichoric and Caftaric Acid-Standardized Echinacea purpurea Root Extracts with Defined Composition and Favorable Biological Properties." PubMed types: Journal Article; source/date: Molecules, 2026 Jul 3; query strands: B3_sdf_assays_thresholds; DOI: 10.3390/molecules31132351. |
| 42454880 | exclude | Non-varicocele human study lacked claim-level relevance | no | Not cited |  | Title/record-level screening identified a human male or reproductive study without varicocele and without claim-level relevance to the revised framework. Screened record: "Automated comet assay analysis using YOLOv5-based deep learning." PubMed types: Journal Article; source/date: Technol Health Care, 2026 Jul 15; query strands: B3_sdf_assays_thresholds; DOI: 10.1177/09287329261466485. |
| 42456353 | exclude | Nonhuman/in-vitro study did not fill a prespecified E4 mechanistic gap | no | Not cited |  | Title/record-level screening identified a nonhuman, ex vivo, or in-vitro model that did not fill a prespecified E4 mechanistic gap in this narrative update. Screened record: "Differential biomarker responses and associated health risks in two pelagic fish species from the Mediterranean Sea, Egypt." PubMed types: Journal Article; source/date: Mar Pollut Bull, 2026 Jul 15; query strands: B3_sdf_assays_thresholds; DOI: 10.1016/j.marpolbul.2026.120098. |
| 42457799 | exclude | Nonhuman/in-vitro study did not fill a prespecified E4 mechanistic gap | no | Not cited |  | Title/record-level screening identified a nonhuman, ex vivo, or in-vitro model that did not fill a prespecified E4 mechanistic gap in this narrative update. Screened record: "Kaempferol-loaded BSA nanoparticles as an alternative cryoprotectant attenuate apoptosis and autophagy in Bovine Spermatogonial Stem Cells (BSSCs) during cryopreservation." PubMed types: Journal Article; source/date: Sci Rep, 2026 Jul 15; query strands: B2_oxidative_stress_male_infertility; DOI: 10.1038/s41598-026-60668-7. |
| 42462956 | exclude | Nonhuman/in-vitro study did not fill a prespecified E4 mechanistic gap | no | Not cited |  | Title/record-level screening identified a nonhuman, ex vivo, or in-vitro model that did not fill a prespecified E4 mechanistic gap in this narrative update. Screened record: "Adaptation and application of the Comet Assay to genotoxicity assessments in elasmobranchs." PubMed types: Journal Article; source/date: Environ Pollut, 2026 Jul 17; query strands: B3_sdf_assays_thresholds; DOI: 10.1016/j.envpol.2026.128797. |
| 42463955 | exclude | Female/ART/perinatal topic outside prespecified couple-level question | no | Not cited |  | Title/record-level screening identified a female, ART, embryo, pregnancy, or perinatal focus outside the prespecified male-varicocele/couple-level question. Screened record: "Is assisted oocyte activation an effective method in poor responders?" PubMed types: Journal Article; source/date: Arch Gynecol Obstet, 2026 Jul 16; query strands: C3_female_factors_outcomes; DOI: 10.1007/s00404-026-08491-3. |
| 42464323 | retain | Retained for detailed assessment | yes | Detailed assessment completed; not cited because the indirect sperm-source comparison does not address varicocele or oocyte repair. |  | Retrospective comparison in 60 men or couples with elevated SDF found no significant cumulative-live-birth or pregnancy differences between fresh and cryopreserved testicular sperm. It is a non-varicocele intervention comparison and does not measure oocyte repair. Screened record: "The impact of fresh versus cryopreserved testicular sperm on in-vitro fertilization outcomes in couples with elevated sperm DNA fragmentation: a retrospective cohort study." PubMed types: Journal Article; source/date: Reprod Biol Endocrinol, 2026 Jul 17; query strands: C2_sdf_art_outcomes; DOI: 10.1186/s12958-026-01594-1. |
| 42466593 | retain | Retained for detailed assessment | yes | Detailed assessment completed; not cited because the hormonal ratio was not studied in varicocele and was not validated against fertility, couple, or reserve outcomes. |  | Among 1,568 men with primary infertility, including 360 with low testosterone, the FSH-to-inhibin-B ratio was associated with severity of gonadal dysfunction. No varicocele, fertility/couple endpoint, or reserve validation was reported. Screened record: "Follicle-Stimulating Hormone to Inhibin B Ratio Among Primary Infertile Men With Low Testosterone-A Biochemical Marker of Testicular Reserve." PubMed types: Journal Article; source/date: Andrology, 2026 Jul 17; query strands: B3_sdf_assays_thresholds, B5_amh_inhibin_b; DOI: 10.1111/andr.70321. |
| 42466650 | exclude | Female/ART/perinatal topic outside prespecified couple-level question | no | Not cited |  | Title/record-level screening identified a female, ART, embryo, pregnancy, or perinatal focus outside the prespecified male-varicocele/couple-level question. Screened record: "Effect of Ejaculatory Abstinence Duration on Laboratory and Clinical Outcomes in IVF: A Retrospective Analysis." PubMed types: Journal Article; source/date: Andrology, 2026 Jul 17; query strands: C2_sdf_art_outcomes; DOI: 10.1111/andr.70322. |
| 42470213 | exclude | Secondary source added no claim-relevant primary or clinical-boundary evidence | no | Not cited |  | PubMed publication type or title identified a review, synthesis, guideline comparison, or committee opinion; it added no claim-relevant primary or clinical-boundary evidence beyond the retained records. Screened record: "Oxidative Stress Induced by Air Pollutants and Its Role in Testicular Dysfunction." PubMed types: Journal Article; Review; source/date: J Appl Toxicol, 2026 Jul 18; query strands: B2_oxidative_stress_male_infertility; DOI: 10.1002/jat.70352. |
| 42470492 | exclude | Nonhuman/in-vitro study did not fill a prespecified E4 mechanistic gap | no | Not cited |  | Title/record-level screening identified a nonhuman, ex vivo, or in-vitro model that did not fill a prespecified E4 mechanistic gap in this narrative update. Screened record: "Investigating the effects of nicotinamide mononucleotide administration on testicular apoptosis, and mitochondrial function in doxorubicin-treated rats." PubMed types: Journal Article; source/date: Mol Biol Rep, 2026 Jul 18; query strands: B2_oxidative_stress_male_infertility; DOI: 10.1007/s11033-026-12391-9. |
| 42470970 | exclude | Secondary source added no claim-relevant primary or clinical-boundary evidence | no | Not cited |  | PubMed publication type or title identified a review, synthesis, guideline comparison, or committee opinion; it added no claim-relevant primary or clinical-boundary evidence beyond the retained records. Screened record: "Applications of artificial intelligence and machine learning in assisted reproductive technology: Focus on In Vitro fertilization (IVF) and intracytoplasmic sperm injection (ICSI)." PubMed types: Journal Article; Review; source/date: Tissue Cell, 2026 Jul 11; query strands: C3_female_factors_outcomes; DOI: 10.1016/j.tice.2026.103777. |
| 42471625 | retain | Retained for detailed assessment | yes | Detailed assessment completed; not cited because the exploratory morphology thresholds were non-varicocele and lacked a reproductive outcome. |  | Single-center analysis of 135 semen samples found only weak correlations between morphology indices and DFI and derived exploratory screening thresholds. It was non-varicocele and had no reproductive endpoint. Screened record: "Sperm morphology indices as exploratory screening tools for elevated DNA fragmentation: a single-centre correlation and threshold derivation study." PubMed types: Journal Article; source/date: BMC Urol, 2026 Jul 18; query strands: B3_sdf_assays_thresholds; DOI: 10.1186/s12894-026-02261-8. |
| 42472777 | exclude | Unrelated keyword collision | no | Not cited |  | The record was retrieved through broad oxidative-stress, repair, or reproduction terms, but title/record-level screening showed an unrelated clinical, organ-system, or laboratory topic. Screened record: "Scorpion envenomation in pregnancy: clinical symptoms, maternal and fetal outcomes and management strategies." PubMed types: Journal Article; source/date: BMC Pregnancy Childbirth, 2026 Jul 20; query strands: C3_female_factors_outcomes; DOI: 10.1186/s12884-026-09576-2. |
| 42474023 | exclude | Secondary source added no claim-relevant primary or clinical-boundary evidence | no | Not cited |  | PubMed publication type or title identified a review, synthesis, guideline comparison, or committee opinion; it added no claim-relevant primary or clinical-boundary evidence beyond the retained records. Screened record: "Molecular and Cellular Pathways and Mechanisms of Antioxidant Compounds Against Oxidative Stress in Obesity-associated Male Infertility: A Comprehensive Review." PubMed types: Journal Article; source/date: Mini Rev Med Chem, 2026 Jul 17; query strands: B2_oxidative_stress_male_infertility; DOI: 10.2174/0113895575455223260702141746. |
| 42478061 | exclude | Nonhuman/in-vitro study did not fill a prespecified E4 mechanistic gap | no | Not cited |  | Title/record-level screening identified a nonhuman, ex vivo, or in-vitro model that did not fill a prespecified E4 mechanistic gap in this narrative update. Screened record: "Antimutagenic and Antigenotoxic Effects of Ruscus aculeatus L. Rhizomes and Roots." PubMed types: Journal Article; source/date: Turk J Pharm Sci, 2026 Jul 21; query strands: B3_sdf_assays_thresholds; DOI: 10.4274/tjps.galenos.2026.59852. |
| 42478660 | retain | Retained for detailed assessment | yes | Cited in the main text as [114] for SDF/DSB measurement discordance in varicocele. | [114] | Study of 70 men with varicocele and 30 controls reported limited agreement among global sperm-DNA-fragmentation and double-strand-break measures across platforms. This supports measurement caution, not a reserve construct or clinical threshold. Screened record: "Multifunctional automated semen analysis system of global SDF and DSBs provides new insights into varicocele diagnosis." PubMed types: Journal Article; source/date: Asian J Androl, 2026 Jul 21; query strands: A1_preserved_fertility, A5_dna_damage_repair, B3_sdf_assays_thresholds; DOI: 10.4103/aja202633. |
| 42479731 | exclude | Female/ART/perinatal topic outside prespecified couple-level question | no | Not cited |  | Title/record-level screening identified a female, ART, embryo, pregnancy, or perinatal focus outside the prespecified male-varicocele/couple-level question. Screened record: "The future of births via medically assisted reproduction in Italy:Scenarios to 2050." PubMed types: Journal Article; source/date: PLoS One, 2026; query strands: C3_female_factors_outcomes; DOI: 10.1371/journal.pone.0354135. |
| 42482624 | exclude | Unrelated keyword collision | no | Not cited |  | The record was retrieved through broad oxidative-stress, repair, or reproduction terms, but title/record-level screening showed an unrelated clinical, organ-system, or laboratory topic. Screened record: "Associations between body mass index and retained placenta in delivery hospitalizations: Analysis of the Nationwide Inpatient Sample, 2016-2020." PubMed types: Journal Article; source/date: Int J Gynaecol Obstet, 2026 Jul 22; query strands: C3_female_factors_outcomes; DOI: 10.1002/ijgo.71214. |
| 42484144 | exclude | Nonhuman/in-vitro study did not fill a prespecified E4 mechanistic gap | no | Not cited |  | Title/record-level screening identified a nonhuman, ex vivo, or in-vitro model that did not fill a prespecified E4 mechanistic gap in this narrative update. Screened record: "SMCP maintains ROS homeostasis during sperm mitochondrial sheath assembly." PubMed types: Journal Article; source/date: Reproduction, 2026 Aug 4; query strands: B2_oxidative_stress_male_infertility; DOI: 10.1093/reprod/xaag090. |
| 42488833 | exclude | Unrelated keyword collision | no | Not cited |  | The record was retrieved through broad oxidative-stress, repair, or reproduction terms, but title/record-level screening showed an unrelated clinical, organ-system, or laboratory topic. Screened record: "Huashi Baidu formula may attenuate adriamycin-induced myocardial injury via modulating NRF2-ATF3-SRXN1 axis-mediated ferroptosis." PubMed types: Journal Article; source/date: Chin Herb Med, 2026 Jul; query strands: B3_sdf_assays_thresholds; DOI: 10.1016/j.chmed.2026.05.009. |
| 42495163 | exclude | Non-varicocele human study lacked claim-level relevance | no | Not cited |  | Title/record-level screening identified a human male or reproductive study without varicocele and without claim-level relevance to the revised framework. Screened record: "Gonadotropin therapy in idiopathic hypogonadal non-obstructive azoospermia (APHRODITE Groups 3-4): a multicenter randomized controlled trial." PubMed types: Journal Article; source/date: Front Reprod Health, 2026; query strands: A9_endocrine_function; DOI: 10.3389/frph.2026.1867412. |
| 42496869 | exclude | Female/ART/perinatal topic outside prespecified couple-level question | no | Not cited |  | Title/record-level screening identified a female, ART, embryo, pregnancy, or perinatal focus outside the prespecified male-varicocele/couple-level question. Screened record: "Fertilization time and oocyte spindle integrity: implication for embryo ploidy." PubMed types: Journal Article; source/date: Reprod Fertil, 2026 Jul 1; query strands: C3_female_factors_outcomes; DOI: 10.1530/RAF-26-0016. |
| 42499284 | exclude | Nonhuman/in-vitro study did not fill a prespecified E4 mechanistic gap | no | Not cited |  | Title/record-level screening identified a nonhuman, ex vivo, or in-vitro model that did not fill a prespecified E4 mechanistic gap in this narrative update. Screened record: "Testicular damage from electromagnetic radiation in rats and evaluation of protective agents." PubMed types: Journal Article; source/date: Urologia, 2026 Jul 25; query strands: B2_oxidative_stress_male_infertility; DOI: 10.1177/03915603261448981. |
| 42503211 | exclude | Nonhuman/in-vitro study did not fill a prespecified E4 mechanistic gap | no | Not cited |  | Title/record-level screening identified a nonhuman, ex vivo, or in-vitro model that did not fill a prespecified E4 mechanistic gap in this narrative update. Screened record: "Chemical Characterization and In Vivo Safety Assessment of Aqueous Extract From Anacardium occidentale L. (Anacardiaceae) Leaves." PubMed types: Journal Article; source/date: Chem Biodivers, 2026 Jul; query strands: B3_sdf_assays_thresholds; DOI: 10.1002/cbdv.71528. |
| 42503275 | exclude | Nonhuman/in-vitro study did not fill a prespecified E4 mechanistic gap | no | Not cited |  | Title/record-level screening identified a nonhuman, ex vivo, or in-vitro model that did not fill a prespecified E4 mechanistic gap in this narrative update. Screened record: "Gestational cadmium exposure triggers POI-Like ovarian dysfunction and accelerated ovarian aging through epigenetic repression of Kl in mice." PubMed types: Journal Article; source/date: Environ Int, 2026 Aug; query strands: C3_female_factors_outcomes; DOI: 10.1016/j.envint.2026.110431. |
| 42510035 | exclude | Non-varicocele human study lacked claim-level relevance | no | Not cited |  | Title/record-level screening identified a human male or reproductive study without varicocele and without claim-level relevance to the revised framework. Screened record: "Development and Preliminary Clinical Evaluation of a Five-Item Prepubertal Risk Questionnaire for Severe Spermatogenic Impairment: A Retrospective Validation Study-Prepubertal Risk Questionnaire for Spermatogenic Impairment." PubMed types: Journal Article; source/date: Diagnostics (Basel), 2026 Jul 11; query strands: A1_preserved_fertility; DOI: 10.3390/diagnostics16142171. |
| 42510056 | exclude | Female/ART/perinatal topic outside prespecified couple-level question | no | Not cited |  | Title/record-level screening identified a female, ART, embryo, pregnancy, or perinatal focus outside the prespecified male-varicocele/couple-level question. Screened record: "Placental Histopathology and Adverse Perinatal Outcomes in ART Pregnancies: A Comparative Analysis of Homologous and Heterologous Techniques." PubMed types: Journal Article; source/date: Diagnostics (Basel), 2026 Jul 14; query strands: C3_female_factors_outcomes; DOI: 10.3390/diagnostics16142192. |
| 42513587 | exclude | Female/ART/perinatal topic outside prespecified couple-level question | no | Not cited |  | Title/record-level screening identified a female, ART, embryo, pregnancy, or perinatal focus outside the prespecified male-varicocele/couple-level question. Screened record: "Effects of Intraovarian Platelet-Rich Plasma on Ovarian Reserve Markers, Oocyte Development, and Pregnancy Outcomes in Women with Diminished Ovarian Reserve: An Uncontrolled Retrospective Before-After Study." PubMed types: Journal Article; source/date: J Clin Med, 2026 Jul 20; query strands: C3_female_factors_outcomes; DOI: 10.3390/jcm15145674. |
| 42515159 | exclude | Nonhuman/in-vitro study did not fill a prespecified E4 mechanistic gap | no | Not cited |  | Title/record-level screening identified a nonhuman, ex vivo, or in-vitro model that did not fill a prespecified E4 mechanistic gap in this narrative update. Screened record: "Oxybenzone (Benzophenone-3) Induces Antioxidant Responses and DNA Strand Breakage in Aiptasia pallida, a Coral Research Model Organism." PubMed types: Journal Article; source/date: Toxics, 2026 Jul 6; query strands: B3_sdf_assays_thresholds; DOI: 10.3390/toxics14070594. |
| 42516232 | retain | Retained for detailed assessment | yes | Retained for Supplementary Table S2 only; not added to the main reference list because female-partner handling and causal limitations do not support a main-text claim. |  | Retrospective observational study of 900 men with varicocele linked dietary or micronutrient status, redox measures, semen quality, and spontaneous conception. The couple endpoint and female-partner handling were insufficient for causal or reserve inference. Screened record: "Dietary antioxidants and micronutrient status in varicocele-associated male infertility: oxidative stress-mediated effects on semen quality." PubMed types: Journal Article; source/date: Front Nutr, 2026; query strands: A1_preserved_fertility, A4_redox_antioxidant, A5_dna_damage_repair, B2_oxidative_stress_male_infertility; DOI: 10.3389/fnut.2026.1832900. |
| 42516294 | exclude | Secondary source added no claim-relevant primary or clinical-boundary evidence | no | Not cited |  | PubMed publication type or title identified a review, synthesis, guideline comparison, or committee opinion; it added no claim-relevant primary or clinical-boundary evidence beyond the retained records. Screened record: "Comparison of Ovarian Stimulation Protocols in Hypogonadotropic Hypogonadism: Case Series and Review of Literature." PubMed types: Case Reports; Journal Article; source/date: Int J Womens Health, 2026; query strands: C3_female_factors_outcomes; DOI: 10.2147/IJWH.S612954. |
| 42516817 | exclude | Female/ART/perinatal topic outside prespecified couple-level question | no | Not cited |  | Title/record-level screening identified a female, ART, embryo, pregnancy, or perinatal focus outside the prespecified male-varicocele/couple-level question. Screened record: "Does the transfer of a poor-quality embryo along with a good-quality embryo influence the intracytoplasmic sperm injection embryo transfer outcomes? A cross-sectional study." PubMed types: Journal Article; source/date: Int J Reprod Biomed, 2026 May; query strands: C3_female_factors_outcomes; DOI: 10.18502/ijrm.v24i5.21289. |
| 42519986 | exclude | Female/ART/perinatal topic outside prespecified couple-level question | no | Not cited |  | Title/record-level screening identified a female, ART, embryo, pregnancy, or perinatal focus outside the prespecified male-varicocele/couple-level question. Screened record: "The Interaction between Embryo Developmental Stage and Endometrial Preparation Protocol on Live Birth Outcomes in Frozen-Thawed Embryo Transfer: A Retrospective Cohort Study." PubMed types: Journal Article; source/date: Gynecol Obstet Invest, 2026 Jul 28; query strands: C3_female_factors_outcomes; DOI: 10.1159/goi/aczag001. |
| 42520101 | exclude | Unrelated keyword collision | no | Not cited |  | The record was retrieved through broad oxidative-stress, repair, or reproduction terms, but title/record-level screening showed an unrelated clinical, organ-system, or laboratory topic. Screened record: "Multi-assay profiling of blood lymphocytes radiosensitivity under protons or photons." PubMed types: Journal Article; source/date: Int J Radiat Biol, 2026 Jul 28; query strands: B3_sdf_assays_thresholds; DOI: 10.1080/09553002.2026.2682849. |
| 42522249 | retain | Retained for detailed assessment | yes | Cited in the main text as [115] as indirect, non-varicocele ART evidence on DFI and cumulative live birth. | [115] | After propensity-score matching, 398 IVF and 398 ICSI cycles were analyzed; cumulative live birth declined across DFI strata after IVF but not after ICSI. This is indirect non-varicocele E3 evidence with a couple-level endpoint and possible residual female or treatment confounding. Screened record: "Differential Effects of Sperm Deoxyribonucleic Acid Fragmentation Index on Cumulative Live Birth Rate per Complete Cycle Following IVF and ICSI Treatment in Patients Without Severe Male Infertility." PubMed types: Journal Article; source/date: Andrology, 2026 Jul 28; query strands: B3_sdf_assays_thresholds, C2_sdf_art_outcomes; DOI: 10.1111/andr.70338. |
| 42523639 | exclude | Female/ART/perinatal topic outside prespecified couple-level question | no | Not cited |  | Title/record-level screening identified a female, ART, embryo, pregnancy, or perinatal focus outside the prespecified male-varicocele/couple-level question. Screened record: "Stage-specific machine learning prediction of cumulative live birth in women with diminished ovarian reserve." PubMed types: Journal Article; source/date: Front Endocrinol (Lausanne), 2026; query strands: C3_female_factors_outcomes; DOI: 10.3389/fendo.2026.1832301. |
| 42523896 | exclude | Nonhuman/in-vitro study did not fill a prespecified E4 mechanistic gap | no | Not cited |  | Title/record-level screening identified a nonhuman, ex vivo, or in-vitro model that did not fill a prespecified E4 mechanistic gap in this narrative update. Screened record: "Levocarnitine mitigates varicocele-induced spermatogenic impairment by restoring mitochondrial integrity and redox homeostasis in rats." PubMed types: Journal Article; source/date: Front Cell Dev Biol, 2026; query strands: A4_redox_antioxidant, A5_dna_damage_repair; DOI: 10.3389/fcell.2026.1783995. |
| 42528043 | exclude | Female/ART/perinatal topic outside prespecified couple-level question | no | Not cited |  | Title/record-level screening identified a female, ART, embryo, pregnancy, or perinatal focus outside the prespecified male-varicocele/couple-level question. Screened record: "Impact of the Etiology of Diminished Ovarian Reserve on Assisted Reproductive Technology Outcomes: A Multicenter Retrospective Cohort Study." PubMed types: Journal Article; Multicenter Study; source/date: J Obstet Gynaecol Res, 2026 Aug; query strands: C3_female_factors_outcomes; DOI: 10.1111/jog.70418. |
| 42531555 | exclude | Unrelated keyword collision | no | Not cited |  | The record was retrieved through broad oxidative-stress, repair, or reproduction terms, but title/record-level screening showed an unrelated clinical, organ-system, or laboratory topic. Screened record: "Caregiver attitudes toward testicular tissue cryopreservation in prepubertal male cancer/HSCT patients A non-metropolitan, Canadian cohort." PubMed types: Journal Article; source/date: Can Urol Assoc J, 2026 Aug; query strands: C3_female_factors_outcomes; DOI: 10.5489/cuaj.9855. |
| 42534779 | exclude | Female/ART/perinatal topic outside prespecified couple-level question | no | Not cited |  | Title/record-level screening identified a female, ART, embryo, pregnancy, or perinatal focus outside the prespecified male-varicocele/couple-level question. Screened record: "Impact of low-level sex chromosome mosaicism (<10%) on clinical outcomes of first fresh embryo transfer: a propensity score-matched retrospective cohort study." PubMed types: Journal Article; source/date: Front Endocrinol (Lausanne), 2026; query strands: C3_female_factors_outcomes; DOI: 10.3389/fendo.2026.1873048. |
| 42539450 | exclude | Female/ART/perinatal topic outside prespecified couple-level question | no | Not cited |  | Title/record-level screening identified a female, ART, embryo, pregnancy, or perinatal focus outside the prespecified male-varicocele/couple-level question. Screened record: "Endometrial thickness is not a risk factor for hypertensive disorders of pregnancy after IVF treatment in women with male-factor infertility." PubMed types: Journal Article; source/date: Front Endocrinol (Lausanne), 2026; query strands: C3_female_factors_outcomes; DOI: 10.3389/fendo.2026.1896764. |
| 42539466 | exclude | Unrelated keyword collision | no | Not cited |  | The record was retrieved through broad oxidative-stress, repair, or reproduction terms, but title/record-level screening showed an unrelated clinical, organ-system, or laboratory topic. Screened record: "Guanxinjing ameliorates coronary microvascular dysfunction in myocardial ischemia-reperfusion injury by alleviating inflammation and restoring endothelial function." PubMed types: Journal Article; source/date: Front Immunol, 2026; query strands: B3_sdf_assays_thresholds; DOI: 10.3389/fimmu.2026.1846953. |
| 42539533 | exclude | Varicocele technical/procedural or otherwise non-germane endpoint | no | Not cited |  | The record addressed a procedural, anatomic, technical, grading, or otherwise nongermane varicocele endpoint and did not inform the revised claims. Screened record: "Density gradient centrifugation versus swim-up for selecting spermatozoa with improved redox activity, calcium status, and DNA integrity in an established rat model of varicocele-induced infertility." PubMed types: Journal Article; source/date: Front Cell Dev Biol, 2026; query strands: A4_redox_antioxidant, A5_dna_damage_repair; DOI: 10.3389/fcell.2026.1880298. |
| 42539562 | exclude | Nonhuman/in-vitro study did not fill a prespecified E4 mechanistic gap | no | Not cited |  | Title/record-level screening identified a nonhuman, ex vivo, or in-vitro model that did not fill a prespecified E4 mechanistic gap in this narrative update. Screened record: "Optimizing stimulation parameters: transpalpebral and transbrain electrical stimulation for retinal protection in RCS rats." PubMed types: Journal Article; source/date: Front Cell Dev Biol, 2026; query strands: B3_sdf_assays_thresholds; DOI: 10.3389/fcell.2026.1853872. |
| 42545011 | exclude | Unrelated keyword collision | no | Not cited |  | The record was retrieved through broad oxidative-stress, repair, or reproduction terms, but title/record-level screening showed an unrelated clinical, organ-system, or laboratory topic. Screened record: "Best Practice and Recommendations for Pregnant Orthopedic Surgeons Part II: Early Career and Practice." PubMed types: Journal Article; source/date: J Am Acad Orthop Surg, 2026 Aug 3; query strands: C3_female_factors_outcomes; DOI: 10.5435/JAAOS-D-25-01522. |
| 42546117 | exclude | Female/ART/perinatal topic outside prespecified couple-level question | no | Not cited |  | Title/record-level screening identified a female, ART, embryo, pregnancy, or perinatal focus outside the prespecified male-varicocele/couple-level question. Screened record: "Mitochondria-targeted therapeutic strategies in assisted reproduction: challenges and potential solutions." PubMed types: Journal Article; source/date: Hum Reprod, 2026 Aug 3; query strands: C3_female_factors_outcomes; DOI: 10.1093/humrep/deag118. |
| 42546152 | exclude | Non-varicocele human study lacked claim-level relevance | no | Not cited |  | Title/record-level screening identified a human male or reproductive study without varicocele and without claim-level relevance to the revised framework. Screened record: "Industrial Hydrocarbons Exposure Deteriorates Sperm Integrity and Reproductive Function." PubMed types: Journal Article; source/date: J Biochem Mol Toxicol, 2026 Aug; query strands: B2_oxidative_stress_male_infertility; DOI: 10.1002/jbt.71052. |
| 42550085 | retain | Retained for detailed assessment | yes | Cited in the main text as [113] for short-term prediction of semen recovery after repair. | [113] | Prospective observational study of 131 men with varicocele and 20 controls; preoperative DFI, abnormal morphology, and progressive motility predicted complete 3-month semen recovery. The endpoint was short-term male laboratory recovery, without external validation or a couple outcome. Screened record: "Multimodal biomarker profiling in varicocele predicts surgical recovery." PubMed types: Journal Article; Observational Study; source/date: Biomark Med, 2026 Jul; query strands: A3_inflammatory_microenvironmental, A5_dna_damage_repair, A9_endocrine_function, A10_predictors_outcomes_after_repair, B3_sdf_assays_thresholds; DOI: 10.1080/17520363.2026.2686307. |
| 42552644 | exclude | Female/ART/perinatal topic outside prespecified couple-level question | no | Not cited |  | Title/record-level screening identified a female, ART, embryo, pregnancy, or perinatal focus outside the prespecified male-varicocele/couple-level question. Screened record: "Infertility and Risk of Congenital Anomalies: A Population-Based Cohort Study." PubMed types: Journal Article; source/date: Paediatr Perinat Epidemiol, 2026 Aug 4; query strands: C3_female_factors_outcomes; DOI: 10.1111/ppe.70184. |
| 42554763 | exclude | Non-varicocele human study lacked claim-level relevance | no | Not cited |  | Title/record-level screening identified a human male or reproductive study without varicocele and without claim-level relevance to the revised framework. Screened record: "Reversible azoospermia associated with the selective JAK1 inhibitor abrocitinib: a case report." PubMed types: Journal Article; source/date: J Assist Reprod Genet, 2026 Aug 5; query strands: B5_amh_inhibin_b; DOI: 10.1007/s10815-026-03993-0. |
| 42558211 | exclude | Secondary source added no claim-relevant primary or clinical-boundary evidence | no | Not cited |  | PubMed publication type or title identified a review, synthesis, guideline comparison, or committee opinion; it added no claim-relevant primary or clinical-boundary evidence beyond the retained records. Screened record: "Endometriosis: surgical management for fertility enhancement." PubMed types: Journal Article; Review; source/date: Front Endocrinol (Lausanne), 2026; query strands: C3_female_factors_outcomes; DOI: 10.3389/fendo.2026.1861188. |
| 42561092 | exclude | Female/ART/perinatal topic outside prespecified couple-level question | no | Not cited |  | Title/record-level screening identified a female, ART, embryo, pregnancy, or perinatal focus outside the prespecified male-varicocele/couple-level question. Screened record: "Ovarian Antral Follicle Toxicity Induced by Phthalate Exposures." PubMed types: Journal Article; source/date: Biol Reprod, 2026 Aug 6; query strands: C3_female_factors_outcomes; DOI: 10.1093/biolre/ioag169. |
| 42566672 | exclude | Non-varicocele human study lacked claim-level relevance | no | Not cited |  | Title/record-level screening identified a human male or reproductive study without varicocele and without claim-level relevance to the revised framework. Screened record: "Seminal Plasma Kisspeptin-1 and Kisspeptin-54 Levels Are Associated With Semen Parameters and Oxidative Stress in Male Infertility." PubMed types: Journal Article; source/date: Clin Endocrinol (Oxf), 2026 Aug 7; query strands: B2_oxidative_stress_male_infertility; DOI: 10.1111/cen.70199. |
| 42566866 | exclude | Female/ART/perinatal topic outside prespecified couple-level question | no | Not cited |  | Title/record-level screening identified a female, ART, embryo, pregnancy, or perinatal focus outside the prespecified male-varicocele/couple-level question. Screened record: "Subserosal and intramural ectopic pregnancy: toward a new classification for improved management." PubMed types: Journal Article; source/date: Eur J Obstet Gynecol Reprod Biol, 2026 Aug 5; query strands: C3_female_factors_outcomes; DOI: 10.1016/j.ejogrb.2026.115355. |
| 42568412 | exclude | Female/ART/perinatal topic outside prespecified couple-level question | no | Not cited |  | Title/record-level screening identified a female, ART, embryo, pregnancy, or perinatal focus outside the prespecified male-varicocele/couple-level question. Screened record: "Recombinant LH priming improves follicular efficiency and pregnancy outcomes in women with diminished ovarian reserve undergoing antagonist IVF stimulation." PubMed types: Journal Article; source/date: Front Endocrinol (Lausanne), 2026; query strands: C3_female_factors_outcomes; DOI: 10.3389/fendo.2026.1877719. |
| 42568462 | exclude | Female/ART/perinatal topic outside prespecified couple-level question | no | Not cited |  | Title/record-level screening identified a female, ART, embryo, pregnancy, or perinatal focus outside the prespecified male-varicocele/couple-level question. Screened record: "Association between ovarian sensitivity index and IVF-ET outcomes in women of advanced maternal age." PubMed types: Journal Article; source/date: Front Endocrinol (Lausanne), 2026; query strands: C3_female_factors_outcomes; DOI: 10.3389/fendo.2026.1889370. |
| 42570055 | exclude | Secondary source added no claim-relevant primary or clinical-boundary evidence | no | Not cited |  | PubMed publication type or title identified a review, synthesis, guideline comparison, or committee opinion; it added no claim-relevant primary or clinical-boundary evidence beyond the retained records. Screened record: "Incomplete male puberty: a new nosographic entity." PubMed types: Journal Article; Review; source/date: Endocrine, 2026 Aug 8; query strands: B5_amh_inhibin_b; DOI: 10.1007/s12020-026-04732-2. |
| 42571040 | exclude | Female/ART/perinatal topic outside prespecified couple-level question | no | Not cited |  | Title/record-level screening identified a female, ART, embryo, pregnancy, or perinatal focus outside the prespecified male-varicocele/couple-level question. Screened record: "Biochemical pregnancy outcomes following dual versus human chorionic gonadotropin-only trigger in gonadotropin-stimulated intrauterine insemination cycles for unexplained infertility: a retrospective cohort study." PubMed types: Journal Article; Comparative Study; source/date: Ann Saudi Med, 2026 Jul-Aug; query strands: C3_female_factors_outcomes; DOI: 10.5144/0256-4947.2026.295. |
| 42571056 | exclude | Non-varicocele human study lacked claim-level relevance | no | Not cited |  | Title/record-level screening identified a human male or reproductive study without varicocele and without claim-level relevance to the revised framework. Screened record: "The Effects of Alpha-Lipoic Acid Supplement on Sperm Parameters and DNA Fragmentation in Couples with Recurrent Pregnancy Loss: A Randomized Controlled Trial." PubMed types: Journal Article; source/date: Int J Fertil Steril, 2026 Jul 27; query strands: B2_oxidative_stress_male_infertility; DOI: 10.22074/ijfs.2026.2055979.1855. |
| 42571583 | exclude | Secondary source added no claim-relevant primary or clinical-boundary evidence | no | Not cited |  | PubMed publication type or title identified a review, synthesis, guideline comparison, or committee opinion; it added no claim-relevant primary or clinical-boundary evidence beyond the retained records. Screened record: "Maximising and Maintaining Fertility in Suspected or Confirmed Endometriosis: A Structured Narrative Review for Clinical Practice." PubMed types: Journal Article; Review; source/date: Cureus, 2026 Jul; query strands: C3_female_factors_outcomes; DOI: 10.7759/cureus.112350. |
| 42571690 | exclude | Secondary source added no claim-relevant primary or clinical-boundary evidence | no | Not cited |  | PubMed publication type or title identified a review, synthesis, guideline comparison, or committee opinion; it added no claim-relevant primary or clinical-boundary evidence beyond the retained records. Screened record: "[Clinical and preprocedural factors influencing in vitro fertilization outcomes: a machine learning-based analysis]." PubMed types: Journal Article; Review; English Abstract; source/date: Orv Hetil, 2026 Aug 9; query strands: C3_female_factors_outcomes; DOI: 10.1556/650.2026.33608. |
| 42574905 | exclude | Nonhuman/in-vitro study did not fill a prespecified E4 mechanistic gap | no | Not cited |  | Title/record-level screening identified a nonhuman, ex vivo, or in-vitro model that did not fill a prespecified E4 mechanistic gap in this narrative update. Screened record: "Cerium MOF encapsulating resveratrol coated PVA for ameliorating high fructose diet induced testicular inflammation and male infertility in rats." PubMed types: Journal Article; source/date: Tissue Cell, 2026 Aug 7; query strands: B2_oxidative_stress_male_infertility; DOI: 10.1016/j.tice.2026.103845. |
| 42575670 | exclude | Female/ART/perinatal topic outside prespecified couple-level question | no | Not cited |  | Title/record-level screening identified a female, ART, embryo, pregnancy, or perinatal focus outside the prespecified male-varicocele/couple-level question. Screened record: "Impact of smoking and antioxidant use on in vitro fertilization success rates: A retrospective cohort study." PubMed types: Journal Article; source/date: Clin Exp Reprod Med, 2026 Aug 11; query strands: B2_oxidative_stress_male_infertility, C3_female_factors_outcomes; DOI: 10.5653/cerm.2025.08781. |
| 42578583 | retain | Retained for detailed assessment | yes | Detailed assessment completed; not cited because it predicts institutional varicocele grade rather than a patient-relevant outcome. |  | Machine-learning study of 3,855 patients used spermatic-vein reflux-time measurements to predict institutional varicocele grade. It reported no patient-relevant fertility or treatment-response outcome, and its grading target raises relevance and circularity concerns. Screened record: "Machine Learning-Assisted Prediction of Varicocele Grade Using Multidimensional Spermatic Vein Reflux Time Analysis." PubMed types: Journal Article; source/date: Andrology, 2026 Aug 11; query strands: A2_hemodynamic_thermal_hypoxic; DOI: 10.1111/andr.70332. |
| 42579653 | exclude | Nonhuman/in-vitro study did not fill a prespecified E4 mechanistic gap | no | Not cited |  | Title/record-level screening identified a nonhuman, ex vivo, or in-vitro model that did not fill a prespecified E4 mechanistic gap in this narrative update. Screened record: "Carissa spinarum L. aqueous root extracts ameliorate oxidative stress-induced infertility in adult male Wistar rats." PubMed types: Journal Article; source/date: PLoS One, 2026; query strands: B2_oxidative_stress_male_infertility; DOI: 10.1371/journal.pone.0355380. |
| 42580659 | exclude | Female/ART/perinatal topic outside prespecified couple-level question | no | Not cited |  | Title/record-level screening identified a female, ART, embryo, pregnancy, or perinatal focus outside the prespecified male-varicocele/couple-level question. Screened record: "A Systematic Assessment of In Vitro Fertilization Add-On Interventions on Canadian Clinic Websites: A Cross-Sectional Study." PubMed types: Journal Article; source/date: J Obstet Gynaecol Can, 2026 Aug 11; query strands: C2_sdf_art_outcomes; DOI: 10.1016/j.jogc.2026.103496. |
| 42582292 | retain | Retained for detailed assessment | yes | Cited in the main text as [116] as indirect, non-varicocele ART evidence on embryo ploidy and euploid-transfer outcome. | [116] | Analysis of 1,410 embryos and 307 euploid-embryo transfer cycles, with maternal confounding addressed, associated paternal age and DFI with embryo aneuploidy but not clinical pregnancy after euploid transfer. This indirect E3 study does not measure oocyte repair. Screened record: "Paternal age and sperm DNA fragmentation independently affect embryonic ploidy but not euploid embryo implantation potential." PubMed types: Journal Article; source/date: Front Endocrinol (Lausanne), 2026; query strands: C2_sdf_art_outcomes; DOI: 10.3389/fendo.2026.1881113. |
| 42582905 | exclude | Secondary source added no claim-relevant primary or clinical-boundary evidence | no | Not cited |  | PubMed publication type or title identified a review, synthesis, guideline comparison, or committee opinion; it added no claim-relevant primary or clinical-boundary evidence beyond the retained records. Screened record: "Varicocelectomy in men with varicocele-associated infertility: a narrative review of semen quality and reproductive outcomes." PubMed types: Journal Article; Review; source/date: Transl Androl Urol, 2026 Jul 31; query strands: A1_preserved_fertility, A5_dna_damage_repair, A10_predictors_outcomes_after_repair; DOI: 10.21037/tau-2026-0189. |
| 42583645 | exclude | Female/ART/perinatal topic outside prespecified couple-level question | no | Not cited |  | Title/record-level screening identified a female, ART, embryo, pregnancy, or perinatal focus outside the prespecified male-varicocele/couple-level question. Screened record: "Risk factors associated with meiotic errors in blastocysts with mosaic results on biopsy." PubMed types: Journal Article; source/date: Hum Reprod Open, 2026; query strands: C3_female_factors_outcomes; DOI: 10.1093/hropen/hoag067. |
| 42586389 | exclude | Unrelated keyword collision | no | Not cited |  | The record was retrieved through broad oxidative-stress, repair, or reproduction terms, but title/record-level screening showed an unrelated clinical, organ-system, or laboratory topic. Screened record: "PRMT6 drives hearing loss by promoting asymmetric arginine dimethylation-mediated FOXG1 degradation and suppressing mitophagy." PubMed types: Journal Article; source/date: Exp Gerontol, 2026 Oct 1; query strands: B3_sdf_assays_thresholds; DOI: 10.1016/j.exger.2026.113278. |
| 42588046 | exclude | Non-varicocele human study lacked claim-level relevance | no | Not cited |  | Title/record-level screening identified a human male or reproductive study without varicocele and without claim-level relevance to the revised framework. Screened record: "Antioxidant Capacity of Seminal Plasma and Its Association with Semen Quality and Dietary Antioxidant Intake." PubMed types: Journal Article; source/date: Nutrients, 2026 Jul 24; query strands: B2_oxidative_stress_male_infertility; DOI: 10.3390/nu18152423. |
| 42590050 | exclude | Non-varicocele human study lacked claim-level relevance | no | Not cited |  | Title/record-level screening identified a human male or reproductive study without varicocele and without claim-level relevance to the revised framework. Screened record: "Sequencing Matters: Comparative Effectiveness of Density-Gradient Centrifugation and MACS for Enhancing Sperm Quality and DNA Integrity." PubMed types: Journal Article; source/date: J Clin Med, 2026 Jul 30; query strands: B3_sdf_assays_thresholds, C2_sdf_art_outcomes; DOI: 10.3390/jcm15155945. |
| 42590222 | exclude | Non-varicocele human study lacked claim-level relevance | no | Not cited |  | Title/record-level screening identified a human male or reproductive study without varicocele and without claim-level relevance to the revised framework. Screened record: "Exploratory Evaluation of Combined Hormonal Indices in Non-Obstructive Azoospermia: Diagnostic Performance and Prediction of TESE Success." PubMed types: Journal Article; source/date: J Clin Med, 2026 Aug 6; query strands: B5_amh_inhibin_b; DOI: 10.3390/jcm15156120. |
| 42590931 | exclude | Nonhuman/in-vitro study did not fill a prespecified E4 mechanistic gap | no | Not cited |  | Title/record-level screening identified a nonhuman, ex vivo, or in-vitro model that did not fill a prespecified E4 mechanistic gap in this narrative update. Screened record: "Ginkgo Biloba Standardized Extract EGb 761 Reduces Light and Toxin-Induced Photoreceptor Cell Damage via Regulating Oxidative Stress and Autophagy." PubMed types: Journal Article; source/date: Curr Eye Res, 2026 Aug 12; query strands: B3_sdf_assays_thresholds; DOI: 10.1080/02713683.2026.2715153. |
| 42591084 | exclude | Female/ART/perinatal topic outside prespecified couple-level question | no | Not cited |  | Title/record-level screening identified a female, ART, embryo, pregnancy, or perinatal focus outside the prespecified male-varicocele/couple-level question. Screened record: "Modified clomiphene citrate protocol versus gonadotropin-releasing hormone antagonist protocol in in vitro fertilization: a propensity score-matched retrospective study on cost and efficacy." PubMed types: Journal Article; Comparative Study; source/date: Front Endocrinol (Lausanne), 2026; query strands: B5_amh_inhibin_b, C3_female_factors_outcomes; DOI: 10.3389/fendo.2026.1888569. |
| 42592830 | exclude | Secondary source added no claim-relevant primary or clinical-boundary evidence | no | Not cited |  | PubMed publication type or title identified a review, synthesis, guideline comparison, or committee opinion; it added no claim-relevant primary or clinical-boundary evidence beyond the retained records. Screened record: "The role of antioxidants in idiopathic male factor infertility." PubMed types: Journal Article; source/date: Curr Opin Urol, 2026 Aug 13; query strands: B2_oxidative_stress_male_infertility, B3_sdf_assays_thresholds; DOI: 10.1097/MOU.0000000000001442. |
| 42593434 | retain | Retained for detailed assessment | yes | Retained for Supplementary Table S2 only; not added to the main reference list because the small uncontrolled couple cohort cannot support a treatment or reserve inference. |  | Retrospective report of 42 couples after failed IVF/ICSI described live birth after varicocele embolization; maternal age was limited to 35 years or younger. There was no comparator and no ovarian-reserve adjustment. Screened record: "Live Birth Rates After Varicocele Embolization in Patients After Failed In Vitro Fertilization With Intracytoplasmic Sperm Injection." PubMed types: Journal Article; source/date: Andrology, 2026 Aug 13; query strands: C3_female_factors_outcomes; DOI: 10.1111/andr.70328. |
| 42593765 | exclude | Secondary source added no claim-relevant primary or clinical-boundary evidence | no | Not cited |  | PubMed publication type or title identified a review, synthesis, guideline comparison, or committee opinion; it added no claim-relevant primary or clinical-boundary evidence beyond the retained records. Screened record: "Association Between Leukocytospermia and Varicocele in Patients With Infertility." PubMed types: Journal Article; Review; source/date: Am J Reprod Immunol, 2026 Aug; query strands: A3_inflammatory_microenvironmental, A4_redox_antioxidant; DOI: 10.1111/aji.70308. |
| 42597427 | exclude | Non-varicocele human study lacked claim-level relevance | no | Not cited |  | Title/record-level screening identified a human male or reproductive study without varicocele and without claim-level relevance to the revised framework. Screened record: "Quantitative analysis of vitamin E, 5-methyltetrahydrofolate, and glutathione in seminal plasma by liquid chromatography-tandem mass spectrometry." PubMed types: Journal Article; source/date: Front Chem, 2026; query strands: B2_oxidative_stress_male_infertility; DOI: 10.3389/fchem.2026.1900122. |
| 42599750 | exclude | Secondary source added no claim-relevant primary or clinical-boundary evidence | no | Not cited |  | PubMed publication type or title identified a review, synthesis, guideline comparison, or committee opinion; it added no claim-relevant primary or clinical-boundary evidence beyond the retained records. Screened record: "The Value of Platelet-Rich Plasma Treatment in Reproductive Disorders: An Umbrella Review." PubMed types: Journal Article; Systematic Review; Review; source/date: Am J Reprod Immunol, 2026 Aug; query strands: C3_female_factors_outcomes; DOI: 10.1111/aji.70305. |
| 42601861 | exclude | Female/ART/perinatal topic outside prespecified couple-level question | no | Not cited |  | Title/record-level screening identified a female, ART, embryo, pregnancy, or perinatal focus outside the prespecified male-varicocele/couple-level question. Screened record: "Determinants of clinical pregnancy outcomes in women with diminished ovarian reserve undergoing repeated assisted reproductive technology cycles: a retrospective cohort analysis of 3,895 oocyte retrieval cycles." PubMed types: Journal Article; source/date: Front Endocrinol (Lausanne), 2026; query strands: C3_female_factors_outcomes; DOI: 10.3389/fendo.2026.1846798. |
| 42601902 | exclude | Female/ART/perinatal topic outside prespecified couple-level question | no | Not cited |  | Title/record-level screening identified a female, ART, embryo, pregnancy, or perinatal focus outside the prespecified male-varicocele/couple-level question. Screened record: "Direct IVF versus prior laparoscopic cystectomy for ovarian endometrioma ≥4 cm: cumulative live birth rate and implications for fertility preservation - a retrospective cohort study." PubMed types: Journal Article; Comparative Study; source/date: Front Endocrinol (Lausanne), 2026; query strands: C3_female_factors_outcomes; DOI: 10.3389/fendo.2026.1851907. |
| 42602828 | exclude | Female/ART/perinatal topic outside prespecified couple-level question | no | Not cited |  | Title/record-level screening identified a female, ART, embryo, pregnancy, or perinatal focus outside the prespecified male-varicocele/couple-level question. Screened record: "From a single child to family planning: determinants of returning to ART to build the desired family in a monocentric Italian cohort." PubMed types: Journal Article; source/date: Hum Reprod Open, 2026; query strands: C3_female_factors_outcomes; DOI: 10.1093/hropen/hoag063. |
| 42605456 | exclude | Secondary source added no claim-relevant primary or clinical-boundary evidence | no | Not cited |  | PubMed publication type or title identified a review, synthesis, guideline comparison, or committee opinion; it added no claim-relevant primary or clinical-boundary evidence beyond the retained records. Screened record: "Adequacy of Sperm Count and Motility following Varicocelectomy in Clinical Varicocele Patients in a Tertiary Health Institution, Nigeria." PubMed types: Journal Article; Review; source/date: Niger Med J, 2026 May-Jun; query strands: A10_predictors_outcomes_after_repair; DOI: not listed. |
| 42606815 | exclude | Secondary source added no claim-relevant primary or clinical-boundary evidence | no | Not cited |  | PubMed publication type or title identified a review, synthesis, guideline comparison, or committee opinion; it added no claim-relevant primary or clinical-boundary evidence beyond the retained records. Screened record: "Preimplantation genetic testing for male factor infertility: lights and shadows." PubMed types: Journal Article; Review; source/date: J Endocrinol Invest, 2026 Aug 17; query strands: B3_sdf_assays_thresholds; DOI: 10.1007/s40618-026-03017-9. |
| 42609584 | exclude | Secondary source added no claim-relevant primary or clinical-boundary evidence | no | Not cited |  | PubMed publication type or title identified a review, synthesis, guideline comparison, or committee opinion; it added no claim-relevant primary or clinical-boundary evidence beyond the retained records. Screened record: "Inflammation and Oxidative Stress in Male Infertility: A Narrative Review." PubMed types: Journal Article; Review; source/date: Reprod Med Biol, 2026 Jan-Dec; query strands: A1_preserved_fertility, A3_inflammatory_microenvironmental, A4_redox_antioxidant, A5_dna_damage_repair, A6_genetic_epigenetic_apoptotic, B2_oxidative_stress_male_infertility; DOI: 10.1002/rmb2.70090. |
| 42611823 | exclude | Non-varicocele human study lacked claim-level relevance | no | Not cited |  | Title/record-level screening identified a human male or reproductive study without varicocele and without claim-level relevance to the revised framework. Screened record: "Time-dependent Deterioration of Sperm Motility and DNA Integrity During Prolonged Semen Exposure." PubMed types: Journal Article; Video-Audio Media; source/date: J Vis Exp, 2026 Aug 7; query strands: B3_sdf_assays_thresholds; DOI: 10.3791/71515. |
| 42612060 | exclude | Secondary source added no claim-relevant primary or clinical-boundary evidence | no | Not cited |  | PubMed publication type or title identified a review, synthesis, guideline comparison, or committee opinion; it added no claim-relevant primary or clinical-boundary evidence beyond the retained records. Screened record: "Changes in Serum Inhibin B Following Orchiopexy for Cryptorchidism: A Systematic Review and Meta-Analysis of Endocrine Recovery." PubMed types: Journal Article; Systematic Review; Video-Audio Media; Meta-Analysis; source/date: J Vis Exp, 2026 Jul 31; query strands: B5_amh_inhibin_b; DOI: 10.3791/70621. |
| 42612088 | exclude | Non-varicocele human study lacked claim-level relevance | no | Not cited |  | Title/record-level screening identified a human male or reproductive study without varicocele and without claim-level relevance to the revised framework. Screened record: "Father-Son Comparison of Reproductive Traits." PubMed types: Journal Article; source/date: Andrology, 2026 Aug 18; query strands: B5_amh_inhibin_b; DOI: 10.1111/andr.70364. |
| 42614050 | exclude | Female/ART/perinatal topic outside prespecified couple-level question | no | Not cited |  | Title/record-level screening identified a female, ART, embryo, pregnancy, or perinatal focus outside the prespecified male-varicocele/couple-level question. Screened record: "Dual effects of anti-Müllerian hormone on ovarian response and fertilization in assisted reproductive technology cycles: a retrospective cohort study." PubMed types: Journal Article; source/date: Obstet Gynecol Sci, 2026 Aug 19; query strands: C3_female_factors_outcomes; DOI: 10.5468/ogs.26089. |
| 42615963 | exclude | Secondary source added no claim-relevant primary or clinical-boundary evidence | no | Not cited |  | PubMed publication type or title identified a review, synthesis, guideline comparison, or committee opinion; it added no claim-relevant primary or clinical-boundary evidence beyond the retained records. Screened record: "Male Infertility in the 2025 WHO Infertility Guideline: A Comparative Analysis With AUA/ASRM, EAU, ESHRE, and NICE Recommendations." PubMed types: Journal Article; Review; source/date: Andrology, 2026 Aug 19; query strands: A4_redox_antioxidant; DOI: 10.1111/andr.70369. |
| 42617331 | exclude | Female/ART/perinatal topic outside prespecified couple-level question | no | Not cited |  | Title/record-level screening identified a female, ART, embryo, pregnancy, or perinatal focus outside the prespecified male-varicocele/couple-level question. Screened record: "EFFORT study: Surgery vs assisted reproductive technology among women with deep endometriosis - A multicenter randomized controlled trial." PubMed types: Journal Article; source/date: Eur J Obstet Gynecol Reprod Biol, 2026 Aug 3; query strands: B5_amh_inhibin_b; DOI: 10.1016/j.ejogrb.2026.115350. |
| 42625880 | exclude | Female/ART/perinatal topic outside prespecified couple-level question | no | Not cited |  | Title/record-level screening identified a female, ART, embryo, pregnancy, or perinatal focus outside the prespecified male-varicocele/couple-level question. Screened record: "Nuclear error phenotypes at the first mitosis predict cleavage-stage outcomes but not blastocyst competence." PubMed types: Journal Article; source/date: Front Reprod Health, 2026; query strands: C3_female_factors_outcomes; DOI: 10.3389/frph.2026.1905720. |
| 42627484 | exclude | Secondary source added no claim-relevant primary or clinical-boundary evidence | no | Not cited |  | PubMed publication type or title identified a review, synthesis, guideline comparison, or committee opinion; it added no claim-relevant primary or clinical-boundary evidence beyond the retained records. Screened record: "Astaxanthin and Male Reproductive Health: Mechanisms, Experimental Evidence, and Clinical Perspectives." PubMed types: Journal Article; Review; source/date: Reprod Sci, 2026 Aug 21; query strands: B2_oxidative_stress_male_infertility; DOI: 10.1007/s43032-026-02177-6. |
| 42629663 | exclude | Varicocele technical/procedural or otherwise non-germane endpoint | no | Not cited |  | The record addressed a procedural, anatomic, technical, grading, or otherwise nongermane varicocele endpoint and did not inform the revised claims. Screened record: "Technical and venographic variations in embolization of primary versus recurrent varicocele: A retrospective single-center study." PubMed types: Journal Article; Comparative Study; source/date: Medicine (Baltimore), 2026 Aug 21; query strands: A2_hemodynamic_thermal_hypoxic, A10_predictors_outcomes_after_repair; DOI: 10.1097/MD.0000000000050342. |
| 42630329 | exclude | Female/ART/perinatal topic outside prespecified couple-level question | no | Not cited |  | Title/record-level screening identified a female, ART, embryo, pregnancy, or perinatal focus outside the prespecified male-varicocele/couple-level question. Screened record: "Pregnancy Outcome in Women Aged 40 Years and Above: A Cross Sectional Study." PubMed types: Journal Article; source/date: J Obstet Gynaecol India, 2026 Aug; query strands: C3_female_factors_outcomes; DOI: 10.1007/s13224-025-02214-5. |
| 42630480 | exclude | Female/ART/perinatal topic outside prespecified couple-level question | no | Not cited |  | Title/record-level screening identified a female, ART, embryo, pregnancy, or perinatal focus outside the prespecified male-varicocele/couple-level question. Screened record: "Effect of Intraovarian Platelet-Rich Plasma Injection on Ovarian Function and Reproductive Outcomes in Patients with Poor Ovarian Response." PubMed types: Journal Article; source/date: J Obstet Gynaecol India, 2026 Aug; query strands: C3_female_factors_outcomes; DOI: 10.1007/s13224-025-02204-7. |
| 42638506 | retain | Retained for detailed assessment | yes | Cited in the main text as [110] for exploratory association-level multi-omics evidence. | [110] | Exploratory pilot of 25 young men with left varicocele, grouped by semen quality; gut-microbiome and serum-metabolomic signatures differed between groups. Supports association only: small cross-sectional sample, with no independent fertility or reserve outcome. Screened record: "Systemic Signatures Differentiating Varicocele-Associated Semen Quality Heterogeneity: An Exploratory Multi-Omics Pilot Study." PubMed types: Journal Article; source/date: Andrology, 2026 Aug 25; query strands: A1_preserved_fertility, A8_omics; DOI: 10.1111/andr.70372. |
| 42638727 | exclude | Unrelated keyword collision | no | Not cited |  | The record was retrieved through broad oxidative-stress, repair, or reproduction terms, but title/record-level screening showed an unrelated clinical, organ-system, or laboratory topic. Screened record: "Electroacupuncture alleviates osteoarthritis and is associated with changes in gut microbiota and steroid biosynthesis-related metabolites." PubMed types: Journal Article; source/date: Front Mol Biosci, 2026; query strands: B3_sdf_assays_thresholds; DOI: 10.3389/fmolb.2026.1769807. |
| 42640276 | exclude | Varicocele technical/procedural or otherwise non-germane endpoint | no | Not cited |  | The record addressed a procedural, anatomic, technical, grading, or otherwise nongermane varicocele endpoint and did not inform the revised claims. Screened record: "Cracking the nutcracker myth: CT-based risk stratification of symptomatic left renal vein Compression." PubMed types: Journal Article; source/date: Abdom Radiol (NY), 2026 Aug 25; query strands: A2_hemodynamic_thermal_hypoxic; DOI: 10.1007/s00261-026-05750-4. |
| 42641210 | exclude | Nonhuman/in-vitro study did not fill a prespecified E4 mechanistic gap | no | Not cited |  | Title/record-level screening identified a nonhuman, ex vivo, or in-vitro model that did not fill a prespecified E4 mechanistic gap in this narrative update. Screened record: "Helium plasma-activated conditioned medium promotes spermatogonial stem cell recovery and redox homeostasis under bleomycin-induced testicular injury in SSCs-sertoli cell co culture." PubMed types: Journal Article; source/date: J Reprod Immunol, 2026 Aug 20; query strands: B2_oxidative_stress_male_infertility; DOI: 10.1016/j.jri.2026.104947. |

# Supplementary Table S2. Unit of analysis, female-partner handling, and permissible inference in audited primary human outcome studies

Pregnancy, time-to-pregnancy, and live birth are couple-level endpoints even when the exposure or predictor is male. Reporting or baseline balance is not statistical adjustment; exclusion of a diagnosed female factor does not establish normal female reproductive capacity. Two update records labeled "not cited" are included only to document their couple-level limitations and disposition.

| **Study** | **Design / population** | **Endpoint and unit** | **Female-factor handling** | **Strongest supported inference** | **Inference removed** | **Access** |
| --- | --- | --- | --- | --- | --- | --- |
| [8] Redmon 2019 | Cross-sectional; 709 partners of ongoing unassisted pregnancies; 56 varicocele | Male semen/testis/hormones; unadjusted couple time-to-pregnancy | Partner data collected; ovarian reserve not modeled; pregnant partners slightly older | Selected achieved-conception couples had male laboratory differences and similar observed unadjusted time-to-pregnancy | Does not establish preserved intrinsic male fertility or absence of a fecundity effect | Full text |
| [9] Gorelick 1993 | Cross-sectional tertiary clinic; 1,099 men with male-factor infertility | Varicocele prevalence; male semen/FSH | Female age >40 excluded; age differed; partial female evaluation; no adjustment | Varicocele more prevalent in selected secondary- than primary-infertility clinic groups | No within-man progression or reserve erosion | Primary text available |
| [10] Lipshultz 1977 | Cross-sectional volunteers and subfertile clinic groups | Male testicular size and sperm density | No female/couple factors | Cross-sectional group differences | No progressive atrophy or fertility decline over time | PubMed abstract |
| [59] Ibis 2025 | Prospective post-repair male cohort; n=135 | Male hormones/semen; exploratory partner pregnancy | Female eligibility/adjustment unverifiable | Postoperative Δ17-OHP correlated with ΔTMSC in hypogonadal men | Not a preoperative reserve or pregnancy predictor; pregnancy comparison nonsignificant | PubMed abstract |
| [65] Simon 2014 | Cross-sectional ART; 215 men/couples; embryos nested | Embryo, implantation, pregnancy; couple/embryo units | Female age stratified; residual female/embryology confounding | Indirect ART association among sperm DNA damage, female age, and outcome | No direct measurement of oocyte repair; not varicocele-specific | PubMed abstract |
| [68] Khalafalla 2021 | Retrospective ICSI; 392 couples | Fertilization, pregnancy, miscarriage, live birth | Major female causes excluded; age/AMH measured; stratification, no multivariable adjustment | Favorable female status associated with outcomes in high-SDF subgroup | No proof of oocyte-mediated repair; not varicocele-specific | PubMed abstract |
| [71] Pinto 1994 | Cross-sectional; 946 men seen for non-infertility complaints | Historical paternity; male testis size | No contemporary female/couple data | Varicocele can coexist with prior paternity | Prior paternity is not current male capacity or future fecundity | PubMed abstract |
| [72] GamalEl Din 2023 | Cross-sectional four male groups; n=24 each | Male semen and seminal CALB2 | No operational fertility definition or female data | Supports a preserved semen/biomarker comparator phenotype | Cannot support proven fertility or couple fecundity | Full text |
| [73] Cocuzza 2008 | Prospective controlled author-defined fertile men; 33 with/81 without varicocele | Male semen, ROS, testis size | Operational fertility definition/female handling unverifiable | No higher ROS or abnormal conventional semen in author-defined fertile sample | No independent male-fecundity or protection inference | PubMed abstract |
| [74] Zargooshi 2007 | Prospective short follow-up; 124 military-referral men | Male sperm concentration/motility | No partner or pregnancy data | Incidental high-grade lesions can coexist with normal conventional semen | Not a fertile cohort; no couple outcome or non-progression claim | PubMed abstract |
| [81] Foroughi 2022 | Cross-sectional mixed-referral cohort; n=109 | Male grade, semen, testis volume; self-reported infertility history | No female evaluation/adjustment | Grade correlated imperfectly with male endpoints | Cannot establish fecundity or male reserve | Full text |
| [82] Cantoro 2015 | Prospective nonrandomized self-selected cohort; n=337 | Male semen/hormones and couple pregnancy | Definite female causes excluded; no patient-level female adjustment | Selected-couple association between embolization and pregnancy | Selection prevents strong causal or male-intrinsic inference | Primary record |
| [84] Sountoulides 2025 | Prospective observational self-selected; 18 surgery/16 observation | Male semen only | Female factor excluded in multidisciplinary clinic | Modest male morphology difference | No pregnancy/fertility effect measured | Full text |
| [89] Sun 2018 | Prospective RCT; bilateral vs unilateral; n=358 | Male semen and couple spontaneous pregnancy | Female age balanced; other adjustment unverifiable | Randomized surgical-extent effect on male semen and couple pregnancy | Pregnancy remains couple-level; balance is not adjustment | PubMed abstract |
| [96] Xiao 2026 | Prospective cohort; n=360; 2-year follow-up | Natural pregnancy/live birth; couple endpoint predicted from male variables | Female/couple covariates not reported in accessible record | Feasibility of male-variable prognostic model for couple live birth | Not validation of male reserve without female covariates and external validation | PubMed abstract |
| [98] Abdel-Meguid 2011 | Concealed nonmasked RCT; 145 analyzed | Couple spontaneous pregnancy; male semen | Female factor excluded; female age ≤35 | Repair increased pregnancy in selected younger couples | Couple treatment effect, not male reserve mechanism | PubMed abstract |
| Update screen; not cited: Fang 2026; PMID 42516232; DOI 10.3389/fnut.2026.1832900 | Observational dietary study; 900 men with varicocele | Male semen and spontaneous conception | Diagnosed female infertility excluded; age/ovarian reserve absent from model | Associative dietary and male-phenotype signal | No causal antioxidant-reserve or isolated couple-benefit inference | Full text |
| Update screen; not cited: Verstandig 2026; PMID 42593434; DOI 10.1111/andr.70328 | Retrospective telephone follow-up; 42 couples after failed IVF/ICSI | Couple live birth after embolization | Maternal age ≤35; no ovarian-reserve adjustment; no comparator | Descriptive post-embolization couple outcomes | No causal treatment-effect or male-specific inference | PubMed abstract |

## S2B. Male-only treatment-response studies

| **Study** | **Male endpoint** | **Permissible use** | **Not supported** |
| --- | --- | --- | --- |
| [35] Zohdy 2011 | Testosterone, semen, IIEF-5 | Male endocrine/sexual response; selection bias | Couple fecundity or reserve |
| [49] Mancini 2004 | TAC and semen | Male biomarker comparison | Fertility or independent reserve |
| [50] Ardestani Zadeh 2019 | Postoperative semen | Male laboratory RCT response | Pregnancy/live birth; not measured |
| [51] Abbasi 2020 | Semen, lipid peroxidation, SDF | Male laboratory RCT response | Pregnancy/live birth; not measured |
| [61] Molinaro 2016 | Adolescent inhibin B/testis volume | Male developmental endpoint | Adult fertility/couple outcome |
| [76] Sakamoto 2008 | Oxidative and DNA endpoints | Male biomarker response | Pregnancy/couple outcome |
| [102] Kaltsas 2024 | Sperm retrieval/return to ejaculate | NOA male surgical endpoint | Semen or pregnancy prediction generally |
| [104,105] Samplaski/Jang | Post-repair semen/TMSC | Male prediction model and external validation | Pregnancy/live birth prediction |

Secondary syntheses [66, 67, 69, 83, 88, 90, 103, 106] are labeled as such in the manuscript. No single female-factor strategy is attributed to all studies within a synthesis; the female-age finding in [69] is treated as a study-level ecological subgroup, not a patient-level interaction.

# Supplementary Table S3. Claim-level evidence-tier distribution and coding rules

One claim unit was one tier-homogeneous proposition cluster within a paragraph, figure legend, or table row. When different propositions in the same block relied on different tiers, the block contributed one unit to each represented tier; repeated same-tier statements within a block were collapsed. Methods, administrative text, definitions without empirical content, recommendation-only text, and proposed future study designs were excluded.

| **Tier** | **Definition used for source/directness coding** | **Interpretive rule** |
| --- | --- | --- |
| E1 | Human varicocele-specific outcome data | Pregnancy/live birth remain couple-level even when the predictor is male |
| E2 | Human varicocele-specific biomarker or male laboratory data | Association does not establish reserve, direction, or causality |
| E3 | Indirect human evidence from non-varicocele populations | Cannot be generalized to human varicocele without explicit qualification |
| E4 | Animal or in-vitro evidence | Scopes a mechanism only; no human individual effect or threshold |
| E5 | Conceptual inference or untested model proposition | Must use hypothesis/association language and cannot guide care |

## S3B. Distribution before and after this revision

| **Census scope** | **E1** | **E2** | **E3** | **E4** | **E5** | **Total** | **E4+E5** |
| --- | --- | --- | --- | --- | --- | --- | --- |
| Baseline narrative (Abstract–Conclusions; legends included) | 19 (13.8%) | 36 (26.1%) | 10 (7.2%) | 9 (6.5%) | 64 (46.4%) | 138 | 73 (52.9%) |
| Post-revision narrative | 19 (13.7%) | 40 (28.8%) | 11 (7.9%) | 9 (6.5%) | 60 (43.2%) | 139 | 69 (49.6%) |
| Baseline table-inclusive (adds Tables 1–4) | 25 (12.6%) | 66 (33.3%) | 18 (9.1%) | 18 (9.1%) | 71 (35.9%) | 198 | 89 (44.9%) |
| Post-revision table-inclusive | 25 (11.8%) | 73 (34.6%) | 19 (9.0%) | 19 (9.0%) | 75 (35.5%) | 211 | 94 (44.5%) |

Judgment rules: semen, molecular, hormonal, and imaging measures were coded E2 unless used as a prespecified reproductive/treatment outcome; biomarker association was separated from its reserve interpretation; guideline recommendations were excluded unless they asserted an empirical outcome; Table 5 was excluded because it contains explicit study proposals. The E1–E5 scheme is not a risk-of-bias or certainty-of-evidence rating.
